# Sema6D forward signaling impairs T cell activation and proliferation in head and neck cancer

**DOI:** 10.1172/jci.insight.166349

**Published:** 2024-02-08

**Authors:** Takashi Hirai, Yujiro Naito, Shohei Koyama, Yoshimitsu Nakanishi, Kentaro Masuhiro, Mayuko Izumi, Tomoki Kuge, Maiko Naito, Yumiko Mizuno, Yuta Yamaguchi, Sujin Kang, Moto Yaga, Yu Futami, Satoshi Nojima, Masayuki Nishide, Takayoshi Morita, Yasuhiro Kato, Takeshi Tsuda, Norihiko Takemoto, Yumi Kinugasa-Katayama, Taiki Aoshi, Jordan Kelly Villa, Kazuo Yamashita, Tomohiro Enokida, Yuta Hoshi, Kazuto Matsuura, Makoto Tahara, Hyota Takamatsu, Yoshito Takeda, Hidenori Inohara, Atsushi Kumanogoh

**Affiliations:** 1 Department of Immunopathology, World Premier International Research Center (WPI), Immunology Frontier Research Center (IFReC),; 2 Department of Otorhinolaryngology-Head and Neck Surgery, Graduate School of Medicine,; 3 Department of Respiratory Medicine and Clinical Immunology, Graduate School of Medicine, and; 4 Department of Immunology and Molecular Medicine, Graduate School of Medicine, Osaka University, Suita, Osaka, Japan.; 5 Division of Cancer Immunology, Research Institute/Exploratory Oncology Research and Clinical Trial Center (EPOC), National Cancer Center, Tokyo, Chiba, Japan.; 6 Integrated Frontier Research for Medical Science Division, Institute for Open and Transdisciplinary Research Initiatives (OTRI),; 7 Department of Advanced Clinical and Translational Immunology, Graduate School of Medicine, and; 8 Department of Immune Regulation, Immunology Frontier Research Center (IFReC), RIMD, Osaka University, Suita, Osaka, Japan.; 9 KOTAI Biotechnologies Inc., Suita, Osaka, Japan.; 10 Department of Pathology, Graduate School of Medicine, and; 11 Department of Cellular Immunology, RIMD, Osaka University, Suita, Osaka, Japan.; 12 Department of Head and Neck Medical Oncology, National Cancer Center Hospital East, Kashiwa, Chiba, Japan.; 13 Department of Head and Neck Surgery, National Cancer Center Hospital East, Kashiwa, Chiba, Japan.; 14 Center for Infectious Diseases for Education and Research (CiDER),; 15 Japan Agency for Medical Research and Development – Core Research for Evolutional Science and Technology (AMED–CREST), and; 16 Center for Advanced Modalities and DDS (CAMaD), Osaka University, Suita, Osaka, Japan.

**Keywords:** Immunology, Oncology, Cancer immunotherapy, Head and neck cancer, T cells

## Abstract

Immune checkpoint inhibitors (ICIs) are indicated for a diverse range of cancer types, and characterizing the tumor immune microenvironment is critical for optimizing therapeutic strategies, including ICIs. T cell infiltration and activation status in the tumor microenvironment greatly affects the efficacy of ICIs. Here, we show that semaphorin 6D (Sema6D) forward signaling, which is reportedly involved in coordinating the orientation of cell development and migration as a guidance factor, impaired the infiltration and activation of tumor-specific CD8^+^ T cells in murine oral tumors. Sema6D expressed by nonhematopoietic cells was responsible for this phenotype. Plexin-A4, a receptor for Sema6D, inhibited T cell infiltration and partially suppressed CD8^+^ T cell activation and proliferation induced by Sema6D stimulation. Moreover, mouse oral tumors, which are resistant to PD-1–blocking treatment in wild-type mice, showed a response to the treatment in *Sema6d*-KO mice. Finally, analyses of public data sets of human head and neck squamous cell carcinoma, pan-cancer cohorts, and a retrospective cohort study showed that *SEMA6D* was mainly expressed by nonhematopoietic cells such as cancer cells, and *SEMA6D* expression was significantly negatively correlated with *CD8A*, *PDCD1*, *IFNG*, and *GZMB* expression. Thus, targeting Sema6D forward signaling is a promising option for increasing ICI efficacy.

## Abstract 2

<p>Sema6D expression is associated with reduced CD8^+^ T cell activation and proliferation in the tumor microenvironment of head and neck cancer.</p>

## Graphical abstract

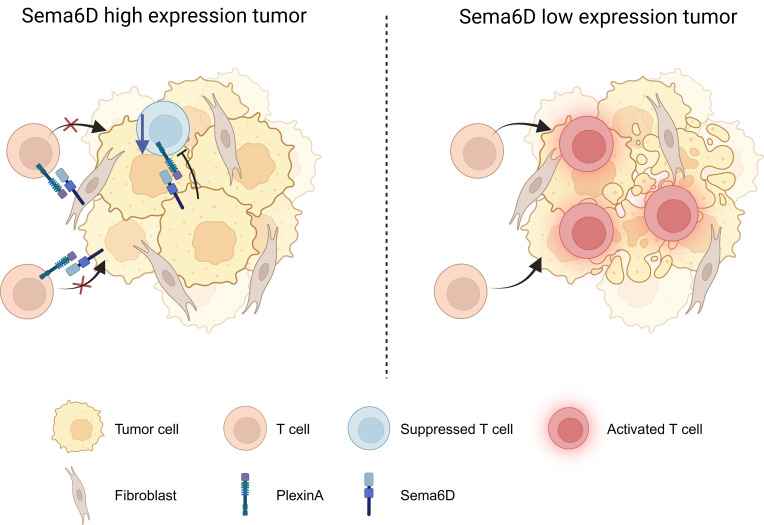

## Introduction

Immunotherapy, as exemplified by immune checkpoint inhibitors (ICIs) such as PD-1–blocking antibodies, has become the standard of care for a wide variety of cancer types, and the immunological profile of the tumor microenvironment (TME) is becoming increasingly important to optimize combination treatment with ICIs (1, 2). Cytotoxic T cells recognize and target tumor-associated antigens, and their infiltration, activation, and proliferation in the TME is essential for the efficacy of ICIs. Tumors that are resistant to immunotherapy have commonly been reported to exhibit low tumor-specific T cell infiltration (3, 4), and are referred to as T cell–noninflamed tumors. In such cancers, targeting T cell suppression mechanisms specific to each TME can improve the therapeutic sensitivity of ICIs by restoring T cell infiltration into tumors. Several groups, including our own, have reported multiple mechanisms for low antitumor CD8^+^ T cell infiltration in the TME, such as upregulation of WNT/β-catenin (5, 6), activation of oncogenic pathways due to gene alterations (7–9), and systemic immunosuppression or cytotoxic T cell depletion caused by tumor localization or the metabolic environment in specific organs (10–13), and targeting these mechanisms significantly improved the therapeutic efficacy of PD-1–blocking treatment in preclinical models.

Semaphorins were originally identified as neural guidance factors that coordinate the orientation of cell development as well as the elongation and migration of diverse cells. Their expression has been detected in various types of immune cells, in which their signaling has been reportedly associated with immune cell migration, differentiation, and effector functions (14, 15). In terms of antitumor immunity, we reported that Sema4A promotes antitumor immunity through CD8^+^ T cell activation (16), and Sema7A interacts with integrin β1 to induce resistance to tyrosine kinase inhibitor treatment (17). Sema6D is a class VI transmembrane-type semaphorin that associates with Plexin-A1 and Plexin-A4, and functions in 2 ways: (a) stimulating receptor Plexin-As as ligands, known as forward signaling (18); and (b) interacting with Plexin-As to induce downstream Sema6D signaling, known as reverse signaling (19). Both types of signaling are involved in tissue morphogenesis and immune responses. Sema6D reverse signaling, which is induced by association with Plexin-A4, regulates lipid metabolism and antiinflammatory polarization in macrophages (20). However, the role of Sema6D in antitumor immune responses and sensitivity to ICIs in the TME is still unknown.

In this study, we developed an oral cancer model using the murine oral squamous cell carcinoma 2 (MOC2) cell line. Tumor progression was significantly decreased in *Sema6d*-knockout (*Sema6d*-KO) mice compared with wild-type (WT) control mice. By investigating bone marrow chimeras using WT and *Sema6d*-KO mice and performing T cell infusion experiments using *Plxna4*-KO cells in *Rag2*-KO mice, we demonstrated that Sema6D expressed by nonhematopoietic cells inhibited the accumulation of CD8^+^ T cells in the tumors, whereas *Plxna4*-KO T cells infiltrated the tumors more than WT T cells. An ex vivo experiment using T cells from tumor-draining lymph nodes (LNs) demonstrated that recombinant Sema6D (rSema6D) inhibited T cell activation and proliferation induced by anti-CD3/anti-CD28 stimulation, and this inhibitory effect was partially decreased in *Plxna4*-KO T cells. An in vivo study indicated that *Sema6d*-overexpressing MOC2 cells were associated with significantly reduced tumor infiltration of CD8^+^ T cells compared with control MOC2 cells. Due to the increased infiltration of CD8^+^ T cells in the *Sema6d*-KO TME, the efficacy of PD-1–blocking therapy against MOC2 was significantly improved in *Sema6d*-KO mice compared with WT mice. Finally, an analysis of public data sets of head and neck squamous cell carcinoma (HNSCC) and pan-cancer cohorts showed that *SEMA6D* was mainly expressed by nonhematopoietic cells such as cancer cells, fibroblasts, and endothelial cells, and *SEMA6D* expression was significantly negatively correlated with the expression of *CD8A*, *PDCD1*, *IFNG*, and *GZMB*. In addition, multiplex immunohistochemistry (IHC) analysis of human HNSCC demonstrated that tumors with low *SEMA6D* expression had significantly higher infiltration of CD8^+^ T cells compared with those with high *SEMA6D* expression. Thus, Sema6D forward signaling might be a vital biomarker for ICI resistance and a promising therapeutic target in a broad range of cancer types, including HNSCC, to improve ICI efficacy by inducing CD8^+^ T cell infiltration into tumors.

## Results

### Tumor progression was significantly reduced in Sema6d-KO mice compared with WT mice, due to increased tumor-infiltrating CD8^+^ T cells.

**Figure 1:**
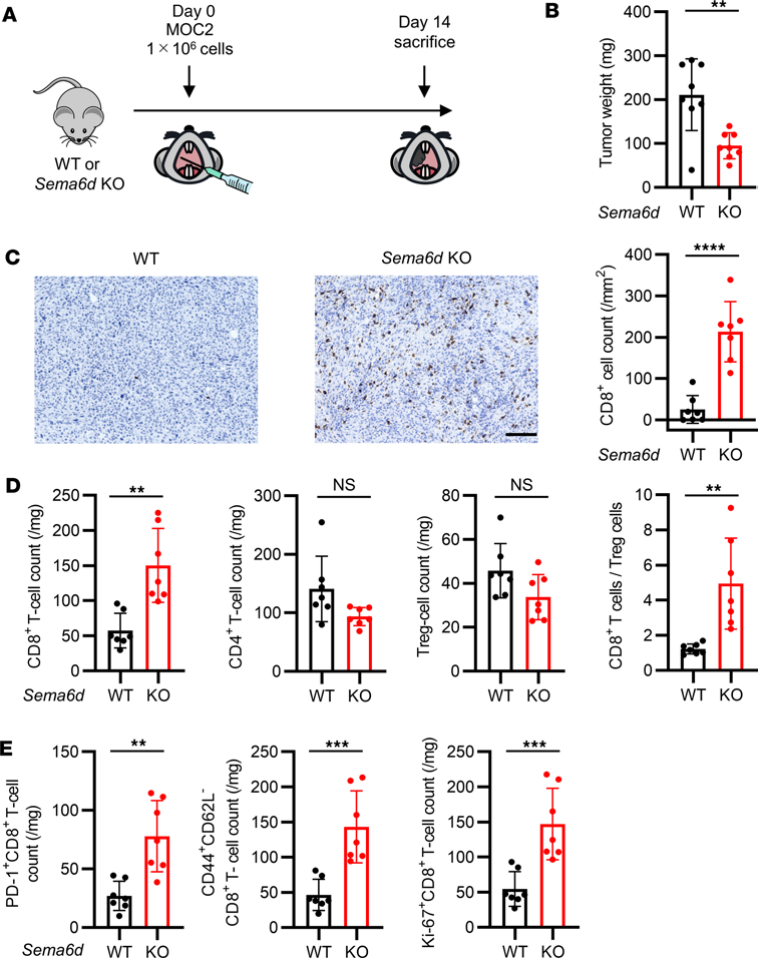
Genetic knockout of *Sema6d* suppressed tumor progression and induced CD8^+^ T cell activation and proliferation in the TME in an oral cancer model. (**A**) Schematic for MOC2 administration and immunological analysis of the TME in a syngeneic oral cancer model. (**B**) Tumor weight in WT (*n* = 8) vs. *Sema6d*-KO mice (*n* = 8) on day 14 after MOC2 injection. (**C**) Representative images of immunohistochemical staining for CD8 (brown) in tumors from WT vs. *Sema6d*-KO mice. Original magnification, ×20. Scale bar: 100 μm. CD8^+^ cell counts in tumors were compared between WT (*n* = 7) and *Sema6d*-KO mice (*n* = 7). (**D**) The following tumor-infiltrating immune cell populations in the TME of WT vs. *Sema6d*-KO mice on day 14 after injection were analyzed by flow cytometry (*n* = 7 per group): CD8^+^ T cells, CD4^+^ T cells, Treg cells, and the ratio of CD8^+^ T cells/Treg cells; and (**E**) PD-1^+^, CD44^+^CD62L^–^ (effector memory), and Ki-67^+^ CD8^+^ T cells. Data in **B**–**E** are representative of 3 independent experiments. **P < 0.01, ***P < 0.001, ****P < 0.0001. Statistical significance determined by 2-tailed Student’s *t* test. Results are presented as mean ± SEM.

To investigate the function of Sema6D in the TME, we initially subcutaneously injected B16F10 melanoma and KP lung cancer cell lines (21) into WT and *Sema6d*-KO mice. However, transplantation of these *Sema6d*-expressing cell lines into *Sema6d*-KO mice could induce an immune reaction to Sema6D as a non-self antigen, leading to tumor shrinkage. Therefore, we screened B6-background cell lines without *Sema6d* expression, and identified the mouse oral carcinoma cell line MOC2 (Supplemental Figure 1A; supplemental material available online with this article; https://doi.org/10.1172/jci.insight.166349DS1). As an orthotopic cancer model, we transplanted MOC2 into the oral mucosa in WT and *Sema6d*-KO mice and observed their clinical course. The tumor growth curve and survival data indicated that tumor growth in *Sema6d*-KO mice was much slower than that in WT mice (Supplemental Figure 1, B and C). To explore the mechanism, we sacrificed mice 14 days after MOC2 transplantation, and analyzed tumor weights and the characteristics of tumor-infiltrating immune cells (Figure 1A). Tumor progression was significantly suppressed in *Sema6d*-KO mice compared with WT mice (Figure 1B). We also analyzed the occurrence of LN metastases in this model. Although LN metastases were significantly more frequent in WT mice compared with *Sema6d*-KO mice, the metastases were more influenced by primary tumor size than by *Sema6d* expression (Supplemental Figure 1D). IHC analysis showed that the frequency of tumor-infiltrating CD8^+^ cells was significantly elevated in *Sema6d*-KO mice compared with WT mice (Figure 1C). We further characterized immune cell populations in the TME by flow cytometry, and found that both the number of CD8^+^ T cells and the ratio of CD8^+^ T cells to regulatory T (Treg) cells was significantly increased in *Sema6d*-KO mice compared with WT mice, whereas the numbers of total CD4^+^ T cells and Treg cells were comparable (Figure 1D). For subsets of CD8^+^ T cells, the numbers of PD-1^+^ cells, effector memory (CD44^+^CD62L^–^) cells, and proliferating (Ki-67^+^) cells were significantly elevated in *Sema6d*-KO mice compared with WT mice (Figure 1E and Supplemental Figure 1E). There were no significant differences between WT mice and *Sema6d*-KO mice regarding the numbers of natural killer (NK) cells (CD3^–^DX5^+^NKp46^+^), B cells (CD3^–^CD19^+^), polymorphonuclear myeloid-derived suppressor cells (PMN-MDSCs; Ly6G^+^CD11b^+^), or monocytic MDSCs (M-MDSCs; Ly6G^–^Ly6C^hi^CD11b^+^) (22) (Supplemental Figure 1F). To compare WT mice and *Sema6d*-KO mice in terms of the steady-state function of immune cells involved in antitumor immunity, we evaluated NK cells in the spleen and also granulocyte macrophage colony–stimulating factor–induced (GM-CSF–induced) dendritic cells using bone marrow cells (GM-DCs). There were no differences in the expression of NK activation markers (Supplemental Figure 2), maturation markers, or immunosuppressive markers, or in the antigen presentation capacity of GM-DCs (Supplemental Figure 3, A and B).

**Figure 2:**
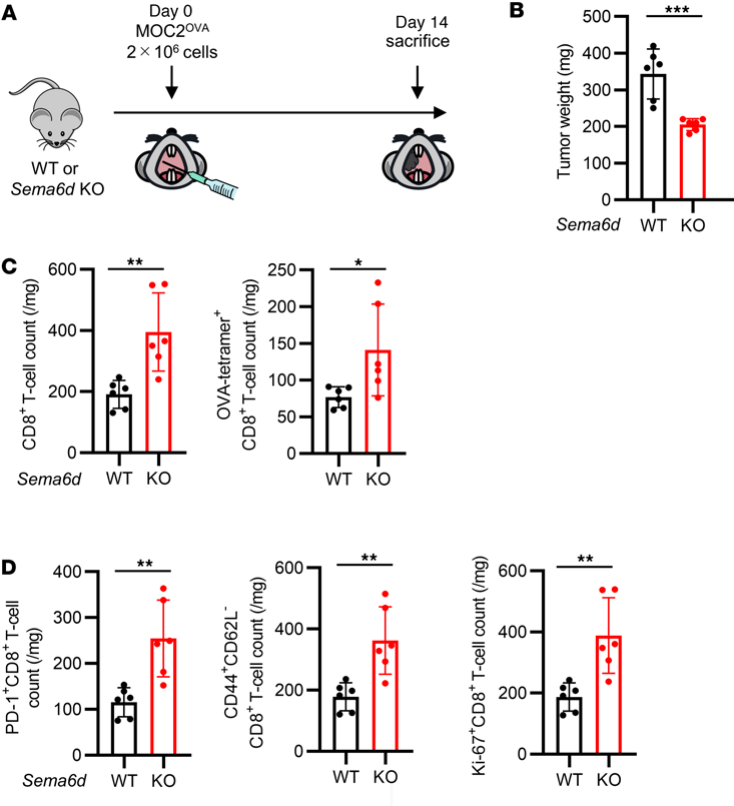
Genetic knockout of *Sema6d* suppressed tumor progression and induced tumor-specific CD8^+^ T cell activation and proliferation in the TME. (**A**) Schematic of OVA-overexpressing MOC2 (MOC2^OVA^) cell administration and immunological analysis of the TME in a syngeneic oral cancer model. (**B**) Tumor weight in WT (*n* = 6) vs. *Sema6d*-KO mice (*n* = 6) on day 14 after MOC2^OVA^ cell injection. (**C**) CD8^+^ T cell counts and OVA-tetramer^+^CD8^+^ T cell counts in the TME, and (**D**) activation and differentiation markers of tumor-infiltrating CD8^+^ T cells in WT (*n* = 6) vs. *Sema6d*-KO mice (*n* = 6) on day 14 after injection were analyzed by flow cytometry. Data in **B**–**D** are representative of 3 independent experiments. *P < 0.05, **P < 0.01, ***P < 0.001. Statistical significance determined by 2-tailed Student’s *t* test. Results are expressed as mean ± SEM.

To further investigate the antitumor T cell response, ovalbumin-overexpressing (OVA-overexpressing) MOC2 (MOC2^OVA^) cells were transplanted into WT mice and *Sema6d*-KO mice (Figure 2A). Compared with WT mice, *Sema6d*-KO mice showed significantly slower tumor growth (Figure 2B) and a significantly higher number of tumor-infiltrating CD8^+^ T cells and OVA-tetramer^+^CD8^+^ T cells (Figure 2C and Supplemental Figure 4). Regarding subsets of CD8^+^ T cells, the numbers of PD-1^+^ cells, effector memory cells, and proliferating cells in *Sema6d*-KO mice were significantly higher than those in WT mice (Figure 2D and Supplemental Figure 4). These results suggest that Sema6D is involved in immune suppression in the TME in an oral cancer model, mainly by acting on CD8^+^ T cells.

### Sema6D expression in nonhematopoietic cells in the TME inhibits the antitumor CD8^+^ T cell immune response.

**Figure 3:**
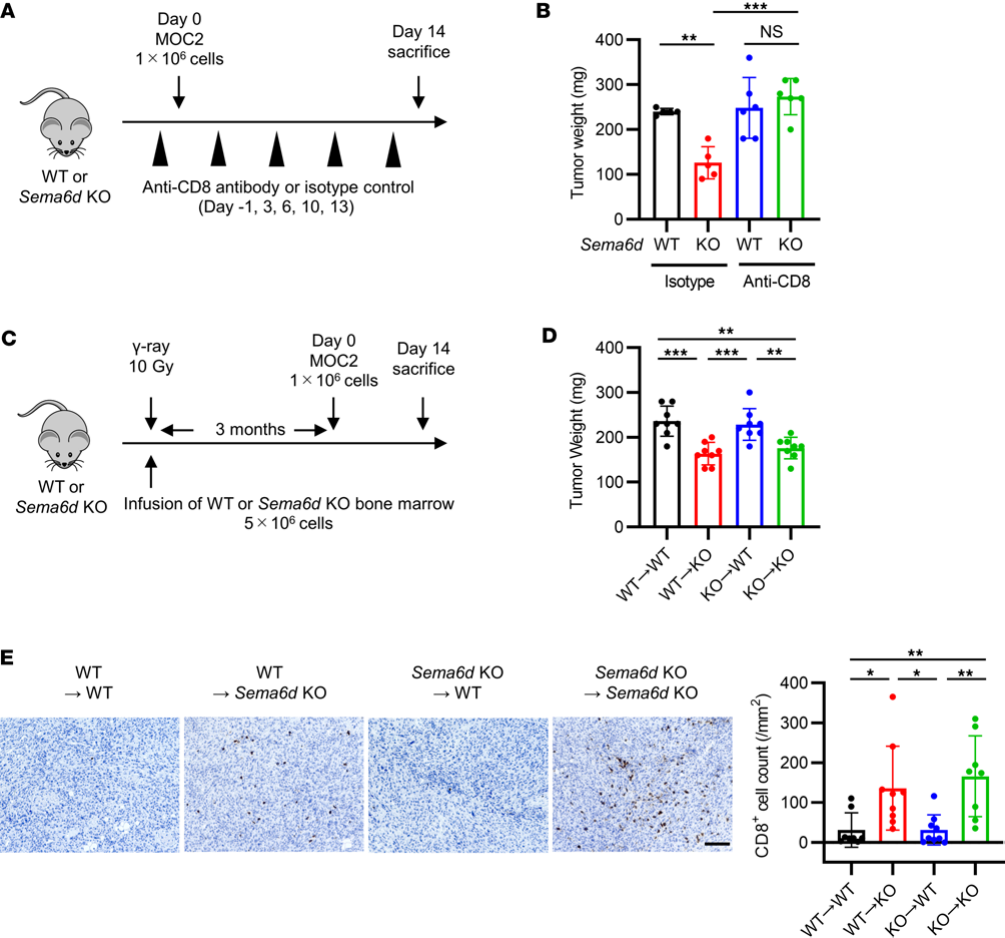
Sema6D expressed by nonhematopoietic cells suppresses CD8^+^ T cell activation and proliferation. (**A**) Schematic of MOC2 cell administration and immunological analysis of the TME in WT and *Sema6d*-KO mice treated with or without CD8^+^ T cell depletion. (**B**) Tumor weight in WT vs. *Sema6d*-KO mice on day 14 after MOC2 injection (*n* = 5–6 per group). Data are representative of 2 independent experiments. (**C**) Schematic of the generation of bone marrow–chimeric mice and immunological analysis after MOC2 cell injection. Bone marrow–chimeric mice were generated by crisscross transplantation of WT or *Sema6d*-KO bone marrow cells into WT or *Sema6d*-KO mice (WT→WT, WT→KO, KO→WT, KO→KO). (**D**) Tumor weight on day 14 after MOC2 cell injection (*n* = 8 per group). Data are representative of 3 independent experiments. (**E**) Representative images of immunohistochemical staining for CD8 (brown) in tumors from WT vs. *Sema6d*-KO mice. Original magnification, ×20. Scale bar: 100 μm. CD8^+^ cell counts in tumors were compared among 4 groups (*n* = 8–9 per group). NS, not significant. *P < 0.05; **P < 0.01; ***P < 0.001. Statistical significance determined by 1-way ANOVA with Tukey’s multiple-comparison test. Results are expressed as mean ± SEM.

To confirm whether CD8^+^ T cells are responsible for inhibiting tumor progression and enhancing activation of the antitumor immune response in *Sema6d*-KO mice, we performed MOC2 transplantation with or without anti-CD8 antibody treatment to deplete CD8^+^ T cells (Figure 3A). CD8 depletion eliminated the differences in tumor growth between WT mice and *Sema6d*-KO mice (Figure 3B).

We then generated bone marrow–chimeric mice by crisscross transplantation of WT or *Sema6d*-KO bone marrow cells into WT or *Sema6d*-KO mice (WT→WT, WT→*Sema6d*-KO, *Sema6d*-KO→WT, *Sema6d*-KO→*Sema6d*-KO) to determine whether the Sema6D that inhibited CD8^+^ T cell activation and proliferation was or was not derived from hematopoietic cells (Figure 3C). Tumor progression was significantly reduced and CD8^+^ cell tumor infiltration was significantly increased in *Sema6d*-KO mice reconstituted with WT or *Sema6d*-KO bone marrow cells (WT→*Sema6d*-KO and *Sema6d*-KO→*Sema6d*-KO) compared with WT mice reconstituted with WT or *Sema6d*-KO bone marrow cells (WT→WT and *Sema6d*-KO→WT) (Figure 3, D and E). These results suggest that nonhematopoietic cell–derived Sema6D in the TME mainly contributes to this phenotype. In addition, total T cells from WT or *Sema6D*-KO mice were transferred to *Rag2*-KO mice, and MOC2 cells were transplanted into these mice (Supplemental Figure 5A). Tumor growth, the number of tumor-infiltrating CD8^+^ T cells (Supplemental Figure 5, B and C), and the subsets of CD8^+^ T cells were comparable between mice that received WT T cells and those that received *Sema6D*-KO T cells (Supplemental Figure 5D), suggesting that Sema6D expressed by T cells is not involved in the immunosuppressive mechanism.

**Figure 4:**
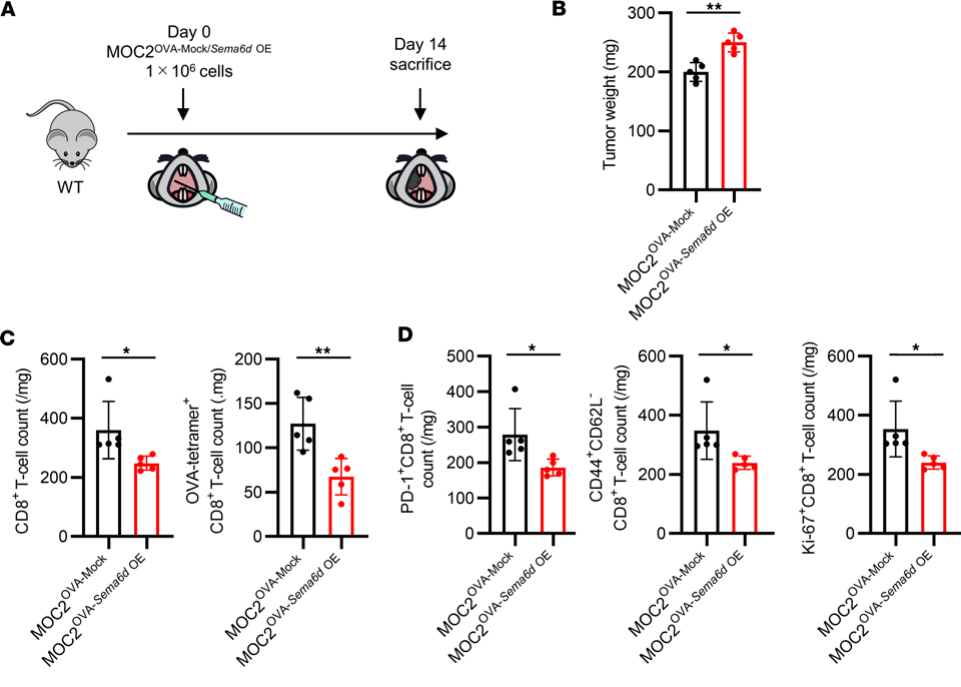
Sema6D expressed by tumor cells inhibits CD8^+^ T cell activation and proliferation in the TME. (**A**) Schematic showing injection of MOC2^OVA-Mock^ or MOC2^OVA-Sema6d^ ^OE^ cells and immunological analysis in WT mice. (**B**) Tumor weight on day 14 after administration of MOC2^OVA-Mock^ or MOC2^OVA-Sema6d^ ^OE^ cells (*n* = 5 per group). (**C**) CD8^+^ T cell counts and OVA-tetramer^+^CD8^+^ T cell counts in the TME, and (**D**) activation and differentiation markers of tumor-infiltrating CD8^+^ T cells on day 14 after injection were analyzed by flow cytometry (*n* = 5 per group). Data in **B**–**D** are representative of 2 independent experiments. *P < 0.05, **P < 0.01. Statistical significance determined by 2-tailed Student’s *t* test. Results are expressed as mean ± SEM.

Although *Sema6d* expression was confirmed in normal oral mucosa, to further investigate how the antitumor immune response was affected by elevated *Sema6d* expression in the TME compared with the normal oral mucosa, we generated a *Sema6d*-overexpressing MOC2^OVA^ cell line (MOC2^OVA-Sema6d^ ^OE^) (Supplemental Figure 6A), and transplanted either MOC2^OVA-Sema6d^ ^OE^ or the corresponding control MOC2^OVA-Mock^ cells into WT mice (Figure 4A). Compared with MOC2^OVA-Mock^ tumors, MOC2^OVA-Sema6d^ ^OE^ tumors showed significantly faster growth and significantly decreased numbers of tumor-infiltrating CD8^+^ T cells and OVA-tetramer^+^ CD8^+^ T cells (Figure 4, B and C, and Supplemental Figure 6B). Regarding subsets of CD8^+^ T cells, the numbers of PD-1^+^ cells, effector memory cells, and proliferating cells were significantly lower in MOC2^OVA-Sema6d^ ^OE^ tumors compared with MOC2^OVA-Mock^ tumors (Figure 4D and Supplemental Figure 6B). To further clarify the contribution of endogenous Sema6D expression to antitumor immunity in tumor cells, we prepared a *Sema6d*-expressing lung cancer KP cell line with OVA (KP^OVA-Mock^) and *Sema6d*-KO KP cell line with OVA (KP^OVA-Sema6d^ ^KO^) (Supplemental Figure 7A), and transplanted both cell lines into the buccal mucosa of WT mice (Supplemental Figure 7B). We then measured tumor weight and performed immune profiling of tumors on day 14. Tumor weight was significantly reduced in KP^OVA-Sema6d^ ^KO^–transplanted mice compared with KP^OVA-Mock^–transplanted mice (Supplemental Figure 7C). Flow cytometry analysis of tumor-infiltrated immune cells showed that the numbers of both CD8^+^ T cells and OVA-specific CD8^+^ T cells were significantly increased in KP^OVA-Sema6d^ ^KO^ tumors (Supplemental Figure 7D). The numbers of PD-1^+^CD8^+^ T cells, CD44^+^CD62L^–^CD8^+^ T cells, and Ki67^+^CD8^+^ T cells were also significantly increased in KP^OVA-Sema6d^ ^KO^ tumors (Supplemental Figure 7E). Consistent with the results in *Sema6d*-overexpression experiments, these results demonstrate that Sema6D expression in nonhematopoietic cells in the TME inhibits antigen-specific CD8^+^ T cell infiltration.

### Sema6D forward signaling through Plexin-A4, a Sema6D receptor, inhibits antitumor CD8^+^ T cell infiltration in the TME.

Since Sema6D derived from nonhematopoietic cells seemed to regulate CD8^+^ T cell function in the TME, we investigated the role of Plexin-A1 and Plexin-A4, which are receptors for Sema6D (19, 20), in T cells isolated from tumor-draining LNs. While *Plxna1* expression was comparable between CD8^+^ and CD4^+^ T cells, *Plxna4* expression was significantly higher in CD8^+^ T cells than CD4^+^ T cells (Supplemental Figure 8A). These results suggest that Sema6D mainly acted on CD8^+^ T cells rather than CD4^+^ T cells. In addition, our previous study showed that macrophage polarization was regulated by the Plexin-A4–Sema6D interaction but not the Plexin-A1–Sema6D interaction (20). Based on these findings, we consider that the Sema6D–Plexin-A4 interaction may play an important role in this immunosuppressive mechanism, particularly in CD8^+^ T cells.

**Figure 5:**
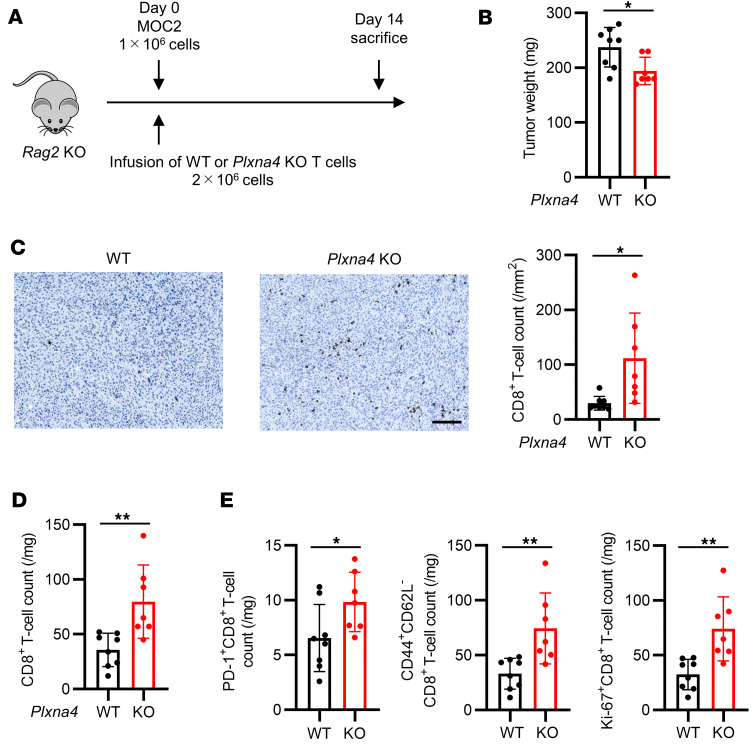
*Plxna4*-deficient CD8^+^ T cells show greater TME infiltration than WT CD8^+^ T cells. (**A**) Schematic of immunological analysis of *Rag2*-KO mice administered WT or *Plxna4*-KO T cells and MOC2 cells. (**B**) Tumor weight on day 14 after administration of WT (*n* = 8) or *Plxna4*-KO T cells (*n* = 7). (**C**) Representative images of immunohistochemical staining for CD8 (brown) in tumors from *Rag2*-KO mice administered WT or *Plxna4*-KO T cells. Original magnification, ×20. Scale bar: 100 μm. CD8^+^ T cell counts were compared between tumors infused with WT (*n* = 8) vs. *Plxna4*-KO T cells (*n* = 7). (**D**) CD8^+^ T cell counts, and (**E**) activation and differentiation markers of tumor-infiltrating CD8^+^ T cells on day 14 after T cell infusion (WT *n* = 8 and *Plxna4*-KO *n* = 7) were analyzed by flow cytometry. Data in **B**–**E** are representative of 2 independent experiments. *P < 0.05, **P < 0.01. Statistical significance determined by 2-tailed Student’s *t* test. Results are expressed as mean ± SEM.

To investigate the role of Plexin-A4 in CD8^+^ T cells, T cells from WT or *Plxna4*-KO mice were transferred to *Rag2*-KO mice along with MOC2 cells (Figure 5A). Compared with mice that received WT T cells, those that received *Plxna4*-KO T cells exhibited significantly slower tumor growth (Figure 5B) and a significantly higher number of tumor-infiltrating CD8^+^ T cells (Figure 5, C and D). Regarding subsets of CD8^+^ T cells, the numbers of PD-1^+^ cells, effector memory cells, and proliferating cells were significantly higher in mice that received *Plxna4*-KO T cells than in those that received WT T cells (Figure 5E and Supplemental Figure 8B). These results suggest that Sema6D derived from nonhematopoietic cells interacts with Plexin-A4 on CD8^+^ T cells, thereby inhibiting the infiltration of CD8^+^ T cells into the TME.

### Sema6D forward signaling inhibits downstream T cell receptor signaling, especially in CD8^+^ T cells, which impairs CD8^+^ T cell activation, effector function, and proliferation.

**Figure 6:**
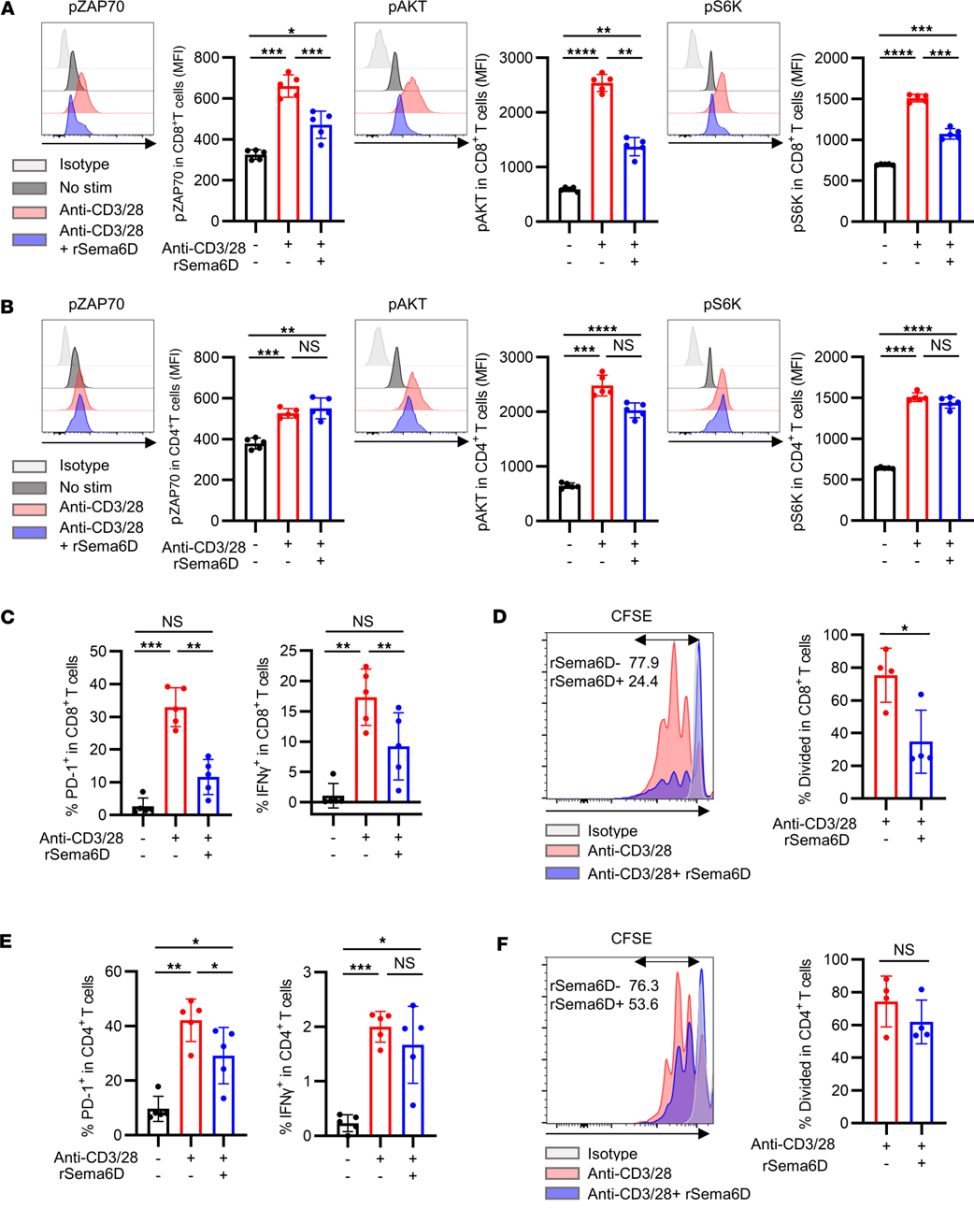
Inhibition of T cell activation, effector function, and proliferation by Sema6D is more pronounced in CD8^+^ T cells than in CD4^+^ T cells. (**A** and **B**) Phosphorylation of ZAP70 (pZAP70), AKT (pAKT), and S6-kinase (pS6K) was compared between CD8^+^ T cells (**A**) and CD4^+^ T cells (**B**) isolated from tumor-draining lymph nodes and stimulated as follows: no stimulation (*n* = 5), anti-CD3/anti-CD28 antibody (48 hours; *n* = 5), and anti-CD3/anti-CD28 antibody plus rSema6D (48 hours; *n* = 5). Representative histograms of p-ZAP70, p-AKT, and p-S6K are shown. Phosphorylation was evaluated by mean fluorescence intensity (MFI). Data are representative of 3 independent experiments. (**C**–**F**) Percentages of CD8^+^ T cells (**C** and **D**) and CD4^+^ T cells (**E** and **F**) isolated from tumor-draining lymph nodes and demonstrating positivity for PD-1, IFN-γ, and CFSE after 48 hours. Representative histograms of cell trace CFSE fluorescence are shown in **D** and **F** (*n* = 4–5 per group). Data are representative of 3 independent experiments. NS, not significant. *P < 0.05; **P < 0.01; ***P < 0.001; ****P < 0.0001. Statistical significance determined by 1-way ANOVA with Tukey’s multiple-comparison test. Results are expressed as mean ± SEM.

To further investigate the mechanism of immunosuppression by Sema6D, we isolated T cells from tumor-draining LNs and stimulated them with anti-CD3/anti-CD28 antibodies with or without rSema6D. We evaluated the phosphorylation levels of molecules involved in T cell receptor (TCR) signaling (phosphorylated Zap70, p-Zap70) and costimulatory signaling (p-Akt and p-S6K). Treatment of CD8^+^ T cells with anti-CD3/anti-CD28 antibodies plus rSema6D significantly reduced the levels of p-Zap70, p-Akt, and p-S6K compared with treatment with anti-CD3/anti-CD28 antibodies alone (Figure 6A). In CD4^+^ T cells, however, the phosphorylation levels of these signal-associated molecules were comparable following treatment with anti-CD3/anti-CD28 antibodies plus rSema6D versus anti-CD3/anti-CD28 antibodies alone (Figure 6B). In addition to analyzing the phosphorylation of proteins involved in TCR and costimulatory signaling, we evaluated T cell activation, effector function, and proliferation by PD-1 expression, IFN-γ production, and the degree of dilution of the dye carboxyfluorescein succinimidyl ester (CFSE), respectively. All of these parameters were significantly reduced in CD8^+^ T cells treated with anti-CD3/anti-CD28 antibodies plus rSema6D compared with those treated with anti-CD3/anti-CD28 antibodies alone (Figure 6, C and D, and Supplemental Figure 9A). In contrast, PD-1 expression was significantly reduced in CD4^+^ T cells treated with anti-CD3/anti-CD28 antibodies plus rSema6D compared with those treated with anti-CD3/anti-CD28 antibodies alone; however, IFN-γ production and cell proliferation were comparable between the 2 groups (Figure 6, E and F, and Supplemental Figure 9A). Moreover, we performed an experiment to evaluate the suppressive effect on CD8^+^ T cells mediated by the interaction between Sema6D and Plexin-A4. MOC2 cells were transplanted into the oral cavity of WT mice and *Plxna4*-KO mice, and T cells collected from their draining LNs were stimulated with anti-CD3/anti-CD28 antibodies with or without rSema6D. Despite the addition of rSema6D, the inhibition of TCR signaling and effector function in CD8^+^ T cells was partially restored in *Plxna4*-KO T cells compared with WT T cells (Supplemental Figure 9B). This suggests that the suppression of T cell activation by rSema6D might be mediated not only through Plexin-A4 but also through Plexin-A1, although an in vivo *Plxna4*-KO T cell transfer experiment demonstrated significant impairment of T cell infiltration and activation, as shown in Figure 5.

Treatment with rSema6D did not alter either NK-activating receptors in NK cells (Supplemental Figure 10A) or maturation markers in GM-DCs (Supplemental Figure 10B). Along with the results discussed above, these data suggest that the immunosuppressive effect of Sema6D is stronger in CD8^+^ T cells than in CD4^+^ T cells, and that the mechanism involves impairment of TCR and costimulatory signaling.

### Inhibition of Sema6D forward signaling improves the efficacy of PD-1–blocking antibody treatment.

**Figure 7:**
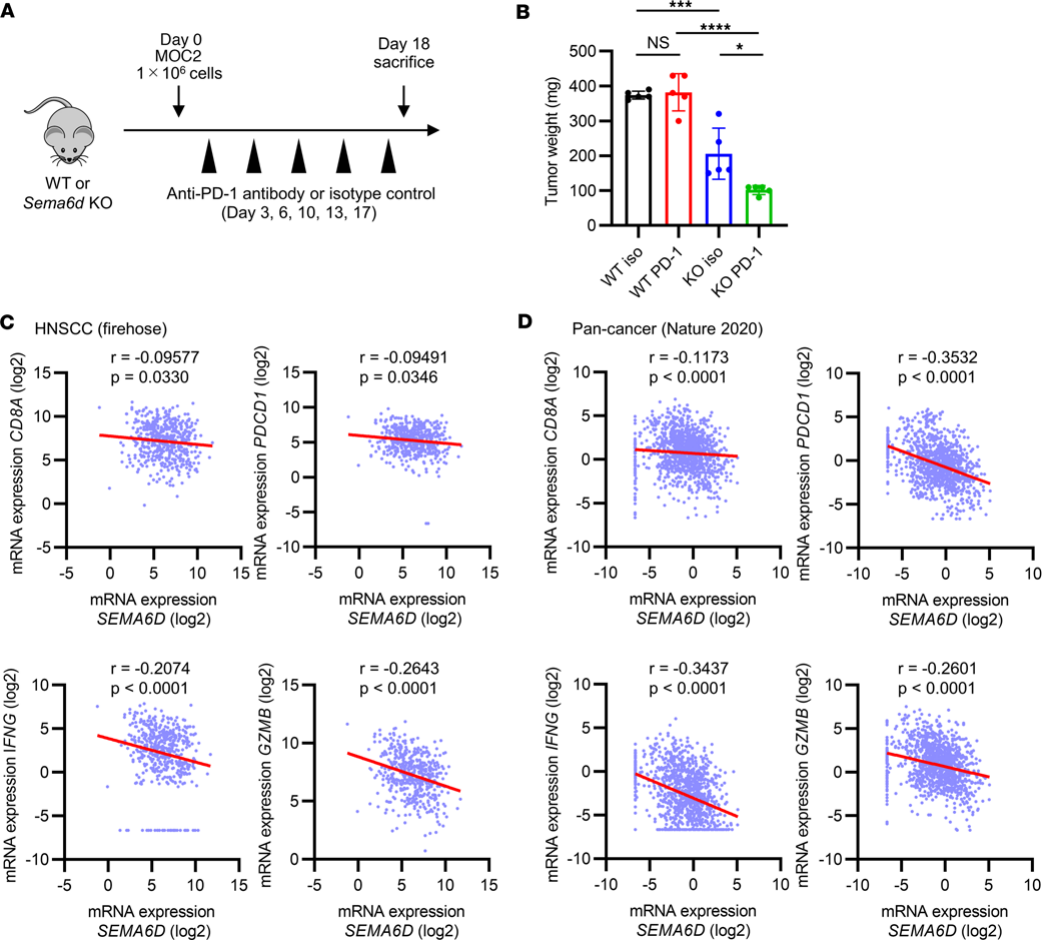
The expression of *SEMA6D* is negatively correlated with genes related to CD8^+^ T cell activation and function in the TME of human cancer. (**A**) Schematic of anti–PD-1 antibody treatment and immunological analysis of WT and *Sema6d*-KO mice administered MOC2 cells. (**B**) Tumor weight on day 18 after administration in WT (*n* = 5) or *Sema6d*-KO mice (*n* = 5). Data are representative of 2 independent experiments. NS, not significant. *P < 0.05; ***P < 0.001; ****P < 0.0001. Statistical significance determined by 1-way ANOVA with Tukey’s multiple-comparison test. Results are expressed as mean ± SEM. (**C** and **D**) Correlation between *SEMA6D* expression and the expression of *CD8A*, *PDCD1*, *IFNG*, and *GZMB* was evaluated in TCGA data sets using Cbioportal; head and neck squamous cell carcinoma (TCGA, Firehose Legacy) (**C**) and pan-cancer analysis of whole genomes (ICGC/TCGA, Nature 2020) (**D**). Correlation was evaluated using Spearman’s rank correlation coefficient. Gene expression of less than 0.01 was evaluated as 0.01.

Since the MOC2 oral cancer model showed that *Sema6d*-KO mice exhibited greater CD8^+^ T cell infiltration in the TME than WT mice, we treated both groups of mice with a PD-1–blocking antibody (Figure 7A). While this treatment did not alter tumor progression in WT mice, it significantly reduced tumor weight in *Sema6d*-KO mice (Figure 7B). These results suggest that loss of Sema6D expression in the TME may improve the sensitivity to ICI treatment.

### SEMA6D expression negatively correlates with that of genes related to cytotoxic T cells and their activation in a public transcriptome data set of patients with cancer.

To investigate the role of Sema6D in human cancer, we used Cbioportal (https://www.cbioportal.org/) to analyze public data sets of bulk RNA sequences from an HNSCC cohort (The Cancer Genome Atlas [TCGA], Firehose Legacy) and a pan-cancer cohort (International Cancer Genome Consortium [ICGC]/TCGA, Nature 2020) (23). We found that the expression of *SEMA6D* was significantly negatively correlated with *CD8A*, *PDCD1*, *IFNG*, and *GZMB* expression, all of which are essential for CD8^+^ T cell activation and function in tumors (Figure 7, C and D). We also analyzed public single-cell RNA sequencing (scRNA-seq) data from patients with oral cancer to determine which cell populations predominantly expressed *SEMA6D* in the TME (24). *SEMA6D* was mainly expressed in the TME by nonhematopoietic cells such as cancer cells, fibroblasts, and endothelial cells, and in normal mucosa it was also expressed by fibroblasts and endothelial cells (Supplemental Figure 11). We next performed multiplex IHC of tumor tissue specimens from patients with HNSCC. Compared with tumors with high *SEMA6D* expression, those with low expression showed significantly increased infiltration of CD8^+^ T cells and PD-1^+^CD8^+^ T cells in the TME (Supplemental Figure 12, A and B). These results suggest that *SEMA6D* expression in human cancers potentially inhibit CD8^+^ T cell activity and infiltration in the TME, including in HNSCC.

## Discussion

This study focused on the function of Sema6D in the TME and its impact on immunological properties and ICI treatment sensitivity. Although several reports have examined the role of Sema6D in cancer, most evaluated the endogenous function of Sema6D in cancer cells. For instance, *SEMA6D* overexpression in human breast cancer cell lines upregulated the expression of genes related to the cell cycle and epithelial-mesenchymal transition, leading to increased malignant characteristics such as migration and invasion (25). In contrast, reduced *SEMA6D* expression was associated both with chemotherapy resistance mediated by regulation of the microRNAs miR-195 and miR-26b and with poor prognosis in breast cancers after chemotherapy, but not after other treatment (26). Thus, this is the first report to our knowledge of a novel immunological function of Sema6D, especially in the TME. Here, we discovered that *Sema6d* deficiency suppressed tumor progression via increased cytotoxic CD8^+^ T cell infiltration into tumors in a murine oral cancer model using MOC2 cells without endogenous *Sema6d* expression. Bone marrow chimera experiments demonstrated that the expression of Sema6D in nonhematopoietic cells was responsible for inhibiting CD8^+^ T cell activation and proliferation partially through Plexin-A4, which was more highly expressed in CD8^+^ T cells than in CD4^+^ T cells. In vitro T cell stimulation by anti-CD3/anti-CD28 antibody with or without rSema6D showed that rSema6D impaired activation, effector function, and proliferation more selectively in CD8^+^ T cells than in CD4^+^ T cells by inhibiting the TCR and costimulatory signaling in tumors.

MOC2 cells, which do not express endogenous *Sema6d*, caused significantly slower tumor progression in *Sema6d*-KO mice compared with WT mice. The importance of *Sema6d* expressed by cancer cells was demonstrated using *Sema6d*-overexpressing MOC2^OVA^ cells, which do not express endogenous *Sema6d*, and KP^OVA^ cells, which naturally express *Sema6d*. Although transcriptome analyses suggest that normal oral mucosa and fibroblasts also express *Sema6d* in mice, the function of Sema6D was not examined in all nonhematopoietic cells in the TME in this study. Therefore, future research using conditional knockout mice should investigate the role of *Sema6d* expressed by cancer-associated fibroblasts and endothelial cells.

A recent study by Celus et al. reported that enhanced Rac1 activation increased the proliferation of *Plxna4*-KO CD8^+^ T cells and improved their ability to migrate to draining LNs and tumors (27). They concluded that Plexin-A4 acted as an immune checkpoint in CD8^+^ T cells, negatively regulating their migration and proliferation through cell-autonomous mechanisms independently of the interaction with host-derived Plexin-A4 ligands. They investigated the expression in the TME of 3 Plexin-A4 ligands, namely Sema3A, Sema6A, and Sema6B, and found that none of them modulated CD8^+^ T cell infiltration into the tumor bed. However, they did not evaluate Sema6D. Sema6D has been shown to interact with both Plexin-A1 and Plexin-A4 during embryonic development, though its binding affinity is stronger with Plexin-A1 than with Plexin-A4 (18). In our data, *Plxna4*-KO CD8^+^ T cells demonstrated an increased ability to infiltrate the TME and partially evaded suppression by the recombinant Sema6D protein. Since the expression of *Plxna4* is higher in CD8^+^ T cells than in CD4^+^ T cells, as shown in both the study by Celus et al. and our own, it is reasonable to assume that T cell suppression by rSema6D occurs more selectively in CD8^+^ T cells than in CD4^+^ T cells. Although further study is needed to investigate the detailed mechanisms of the association between Sema6D and Plexin-A4, our findings and those of Celus et al. demonstrate that both Sema6D, expressed in the TME, and Plexin-A4, expressed by CD8^+^ T cells, may be involved in the T cell noninflamed phenotype.

Finally, we evaluated TCGA data sets of HNSCC and pan-cancer patients using Cbioportal (https://www.cbioportal.org/), and found that *SEMA6D* expression negatively correlated with *CD8A*, *PDCD1*, *IFNG*, and *GZMB* expression, suggesting that the findings in mouse models can be applied to human patients with cancer. Furthermore, we analyzed public scRNA-seq data from patients with oral cancer (24) and found that *SEMA6D* was mainly expressed by nonhematopoietic cells in the TME. In addition, multiplex IHC using tumor tissue specimens from patients with HNSCC showed that *SEMA6D* expression potentially inhibits CD8^+^ T cell infiltration into the TME.

Although therapeutic experiments could not be performed in mice due to the absence of therapeutic Sema6D-blocking antibodies, our data suggest that Sema6D may be a novel therapeutic target to improve the efficacy of immunotherapies, and Sema6D expression in the TME might be a biomarker for predicting the efficacy of ICIs in diverse cancers, including HNSCC.

## Methods

### Mouse study.

C57BL/6J mice were purchased from CLEA Japan. *Sema6d*-KO mice were generated on the C57BL/6J background (28). *Plxna4*-KO mice were provided by Hajime Fujisawa (Nagoya University, Nagoya, Japan) (29). Littermates generated from *Sema6d*-KO and *Plxna4*-KO mice were used as controls. All mice were maintained in specific pathogen–free conditions at the Institute of Experimental Animal Sciences, Osaka University.

### Cell lines.

Mouse lung cancer cell lines were established from lung tumor nodules from mice with mutated *Kras*^G12D^ and homozygous *Tp53* deletion, as previously described (21), and are referred to as KP cell lines. These cell lines were cultured in RPMI 1640 (Nacalai Tesque) with 10% FBS, penicillin (100 U/mL), and streptomycin (100 mg/mL). A mouse melanoma cell line (B16F10) was purchased from ATCC and cultured according to the manufacturer’s protocol. The mouse oral squamous cell carcinoma 2 (MOC2) cell line was purchased from Kerafast, and cultured according to the manufacturer’s protocol. OVA-overexpressing MOC2 (MOC2^OVA^) and OVA-overexpressing KP (KP^OVA^) cells were generated using the pMX retroviral vector system, as previously described (30, 31). In brief, the full OVA segment was amplified by PCR and cloned into a pMX retroviral vector at the BamHI and SalI restriction sites. Retroviral supernatants were generated by transfecting the retroviral packaging vector and each pMX vector containing the gene of interest into the 293T cell line. After transduction with 8 μg/mL polybrene, single-cell-derived clones were obtained by limiting dilution. The expression of OVA was confirmed by IFN-γ production after coculture, with the splenocytes from an OT-1 mouse as an indicator. *Sema6d*-overexpressing MOC2^OVA^ (MOC2^OVA-Sema6d^ ^OE^) cells were generated using the lentiviral vector system. The *Sema6d*-encoded (NM_199241.3) lentiviral vector was purchased (Vector Builder) and MOC2^OVA^ was transfected with lentiviral vectors containing *Sema6d*. *Sema6d*-KO KP^OVA^ (KP^OVA-Sema6d^ ^KO^) cells were generated using the lentiviral vector system. KP^OVA^ was transfected with a CRISPR/Cas9 lentiviral vector targeting *Sema6d* (NM_199241.3) (Vector Builder). The expression of Sema6D was checked by quantitative PCR (32).

### IHC staining and image analysis.

CD8 staining of mouse tumors from oral cancer models was performed by the Applied Medical Research Laboratory using an anti–mouse CD8a antibody (clone D4W2Z, Cell Signaling Technology). Sections were counterstained with hematoxylin and observed by microscopy. The number of infiltrating CD8^+^ cells per mm^2^ in tumor tissue was counted with Hybrid Cell Count (KEYENCE).

### Preparation of bone marrow–derived DCs.

The tibias and femurs of C57BL/6J WT mice or *Sema6d*-KO mice were removed under sterile conditions. Both ends of the bone were cut off with scissors and the bone marrow was rinsed out of the cavity into a sterile culture dish with 2% FBS in PBS. After red blood cell (RBC) lysis, the pelleted cells were washed with 2% FBS in PBS. GM-DCs were generated by culturing bone marrow cells with 20 ng/mL GM-CSF (R&D Systems) in complete RPMI 1640 for 6–8 days as previously described (33).

### OT-1 CD8^+^ T cell proliferation assay.

T cells from splenocytes of OT-1 TCR-Tg mice were isolated using a MojoSort Mouse CD3 T Cell Isolation Kit (BioLegend), and stained with 0.5 μM CFSE (Thermo Fisher Scientific). In the in vitro assay, these cells were cocultured with GM-DCs in a round-bottom 96-well plate in the presence of 10 μg/mL OVA peptides (Sigma-Aldrich), which are recognized by OT-1 lymphocytes. After a 72-hour incubation, flow cytometry was performed with T cell marker staining to analyze the CFSE dilution following each cell division.

### Immune cell isolation from murine tumor samples and analysis.

Tumor-bearing mice were sacrificed, and resected tumors were shredded into small pieces and dissociated with a gentleMACS Octo Dissociator with heaters (Miltenyi Biotec). The program 37C_m_TDK_2 was utilized, and the buffer consisted of 100 U/mL collagenase type IV (Invitrogen), 50 μg/mL DNase I (Roche), and 10% FBS in RPMI 1640 medium. After incubation, cells were treated with RBC lysis buffer (Thermo Fisher Scientific) and passed through a 100-μm cell strainer to remove debris. The cell pellet was suspended by 2% FBS in PBS and used for flow cytometry analysis. Isolated cells were initially stained with the LIVE/DEAD Fixable Dead Cell Stain Kit (Invitrogen). Cells were subsequently incubated with Fc-blocking antibody (BioLegend), and then stained with monoclonal antibodies for several surface and intracellular antigens. Antibody clone numbers for immune analysis are listed in the Supplemental Methods. Foxp3/Transcription Factor Staining Buffer (eBioscience) was used for intracellular staining. Acquisition of samples was performed on a FACSCanto II cytometer (BD Biosciences) equipped with Diva software and analyzed using FlowJo software (BD Biosciences).

The representative plots and gating strategies are shown in the supplemental material.

### Mouse treatment studies.

Murine oral mucosa was inoculated with 5 × 10^5^ MOC2 cells for survival analysis, and 1 × 10^6^ MOC2 cells, 1 × 10^6^ MOC2^OVA-Mock^ cells, 1 × 10^6^ MOC2^OVA-Sema6d^ ^OE^ cells, 2 × 10^6^ MOC2^OVA^ cells, 5 × 10^6^ KP^OVA-Mock^ cells, and 5 × 10^6^ KP^OVA-Sema6d^ ^KO^ cells for immune profiling and the analysis of tumor growth curves and treatment effects. For CD8^+^ T cell depletion experiments, anti-CD8 antibody (clone 53-6.7, BioXCell; 200 μg/mouse) and isotype control antibody (rat IgG2a, BioXCell; 200 μg/mouse) were injected intraperitoneally on days –1, 3, 6, 10, and 13 after tumor inoculation. For anti–PD-1 antibody treatment, anti–PD-1 antibody (clone RMP1-14, BioXCell; 200 μg/mouse) and isotype control antibody (rat IgG2a, BioXCell; 200 μg/mouse) were injected intraperitoneally on days 3, 6, 10, 13, and 17 after tumor inoculation.

### Bone marrow chimeras.

Bone marrow cells were collected from the tibias and femurs of WT or *Sema6d*-KO donor mice using the methods described above. Cells were suspended in 2% FBS in PBS. WT or *Sema6d*-KO recipient mice were irradiated with 10 Gy of gamma rays, and the next day, 5 × 10^6^ T cells in a 100 μL volume were intravenously injected into the tail vein (20). Four types of chimeras were generated: WT→WT; WT→*Sema6d*-KO; *Sema6d*-KO→*Sema6d*-KO; and *Sema6d*-KO→WT.

### T cell infusion into Rag2-KO mice.

T cells collected from the spleen of WT, *Sema6d*-KO, or *Plxna4*-KO mice were isolated using a MojoSort Mouse CD3 T Cell Isolation Kit. A total of 2 × 10^6^ T cells in 100 μL PBS were intravenously injected into the tail vein of *Rag2*-KO mice. Murine oral mucosa was inoculated with MOC2 cells on the same day as T cell infusion.

### In vitro stimulation of T cells.

T cells were isolated from tumor-draining LNs of tumor-bearing mice using a MojoSort Mouse CD3 T Cell Isolation Kit. For in vitro stimulation of effector cells, cells were cultured in RPMI 1640 medium supplemented with 10% FBS, penicillin (100 U/mL), and streptomycin (100 mg/mL), and stimulated for 2 days with plate-bound anti-CD3ε antibody (1 μg/mL; clone 2C11, BD Pharmingen), anti-CD28 antibody (1 μg/mL; clone 37.51, BD Pharmingen), and recombinant mouse Sema6D Fc (10 μg/mL; R&D Systems) or mouse IgG2A Fc (2.5 μg/mL; R&D Systems). Antibodies and their clone numbers for immune analysis are listed in the Supplemental Material.

### In vitro stimulation of NK cells and DCs.

NK cells were isolated from splenocytes, and DCs were generated from bone marrow. For in vitro stimulation, NK cells and DCs were stimulated for 24 hours with plate-bound recombinant mouse Sema6D Fc (10 μg/mL; R&D systems) or mouse IgG2A Fc (2.5 μg/mL; R&D systems) as control. DCs were cultured in RPMI (Nacalai Tesque) supplemented with 10% FBS, penicillin (100 U/mL), and streptomycin (100 mg/mL), and NK cells were cultured in the same medium with the addition of murine IL-2 (100 U/mL; R&D Systems).

### Clinical samples.

Formalin-fixed, paraffin-embedded (FFPE) specimens derived from biopsy samples and surgically resected tumors of patients with HNSCC were investigated in this study. All donors provided written informed consent before sample collection.

### Quantitative real-time PCR.

For all mouse samples, total RNA was extracted with the FastGene RNA Premium Kit (NIPPON Genetics), and for tumor samples from patients with HNSCC, total RNA was extracted with an RNeasy Mini Kit (Qiagen). cDNA was synthesized with SuperScript IV Reverse Transcriptase (Invitrogen). Quantitative PCR reactions were established using TaqMan Fast Advanced Master Mix (Applied Biosystems) and run on QuantStudio 7 (Applied Biosystems). The following primers were used: Plexin-A1 (*Plxna1*; Mm00501110_m1, Thermo Fisher Scientific), Plexin-A4 (*Plxna4*; Mm00558881_m1, Thermo Fisher Scientific), Sema6D (*Sema6d*; Mm00553142_m1, Thermo Fisher Scientific), and endogenous control gene *Actb* (4352341E, Applied Biosystems) for mice, and *SEMA6D* (*SEMA6D*; Hs00227965_m1, Thermo Fisher Scientific) and endogenous control gene *ACTB* (Hs99999903_m1, Thermo Fisher Scientific) for humans. The gene expression data were normalized to the expression of *Actb* or *ACTB*.

### Multiplex IHC and image analysis.

Multiplex IHC and image analysis were performed as previously reported (16). FFPE sections were incubated for 30 minutes at 60°C. Slides were deparaffinized with xylene and rehydrated with a series of graded ethanol solutions in deionized water, and endogenous peroxidase was blocked with 0.3% hydrogen peroxide. Antigen retrieval was performed in Target Retrieval Solution, pH 9 (Dako), in a microwave oven until boiling, and then in a steamer for 20 minutes at 100°C. Primary antibodies targeting PD-1, CD8, CD3, and cytokeratin were paired with the following fluorophores: Opal 570, Opal 620, Opal 690, and Opal 780, respectively, each diluted in 1× Plus Amplification Diluent (Akoya Biosciences). For multiplex staining, the primary antibodies were successively applied to the sections in 6 iterative immunostaining steps. Each step started with retrieving heated antigen using either Citrate Buffer (pH 6.0, Sigma-Aldrich) or Target Retrieval Solution (pH 9.0, Dako) for 20 minutes. Subsequently, each slide was stained with the diluted primary antibodies for 30 minutes. 1× Opal Anti–mouse/rabbit HRP (Akoya Biosciences) was applied as a secondary label with an incubation time of 10 minutes. Antibody signals were visualized after incubating the slides for 10 minutes using the corresponding Opal fluorophore. After 6 rounds of immunostaining, nuclei were counterstained with DAPI and slides were mounted with ProLong Diamond Antifade Mountant with DAPI (Life Technologies). Slides of whole-tissue sections were scanned using a PhenoIMAGER Fusion system (version 1.0.6., Akoya Biosciences). Five areas containing T cells were acquired for each slide. Signals were unmixed and images were exported with InForm (version 2.8, PerkinElmer). Digital quantification of the immune cells was performed using HALO image analysis software, version 3.6.4134 (Indica Labs). Positively stained cells were quantified using the HighPlex FL algorithm version 4.0.4. The density of immune cells was calculated as the number of stained cells divided by the tissue area (per mm^2^).

### Analysis of SEMA6D expression using public HNSCC scRNA-seq data.

10× Chromium scRNA-seq data of HNSCC primary cancer tissue and nontumoral surrounding normal tissue from 23 human patients were obtained from the NCBI Gene Expression Omnibus (GEO GSE181919) (24). The provided unique molecular identifier (UMI) count table and metadata were processed in R (version 4.1.2) using Seurat (version 4.3.0) (34) using the same methods described in the original paper (24). Briefly, the prefiltered Seurat Object UMI count data were log-normalized (Seurat: NormalizeData function) and scaled (Seurat: ScaleData function). Batch effects were corrected using Harmony (harmony: RunHarmony function, group.by.vars = sample.id) (35) before dimensional reduction using UMAP (Seurat: RunUMAP function, dims = 1:50), as described in the original paper (24). Utilizing the original sample (sample.id) and cell type (cell.type) identification provided within the metadata, the log-normalized *SEMA6D* expression was plotted for the cancer (CA) and normal mucosal (NL) tissues (tissue.type) using ggplot2 (version 3.4.1, geom_point + geom_violin; https://ggplot2.tidyverse.org).

### Statistics.

All statistical analyses were performed in GraphPad Prism 8. Survival analysis was performed using Kaplan-Meier survival plots, and the log-rank test *P* value was calculated. Paired and unpaired 2-tailed *t* tests were used for 2-group comparisons, and 1-way ANOVA with Tukey’s multiple-comparison test was used for comparisons of more than 2 groups. Correlation was evaluated using Spearman’s rank correlation coefficient. Numerical data are presented as mean ± SEM. *P* values of less than 0.05 were considered significant.

### Study approval.

The application of animal experiments was approved by the ethical board of Osaka University Graduate School of Medicine (no. 02-051-008). All experiments were performed in accordance with the regulations of Osaka University. The human study was approved by the institutional review board of the National Cancer Center (no. 2015-180) and was conducted in accordance with all relevant ethical guidelines, including the Declaration of Helsinki.

### Data availability.

Values for data points shown in each graph are available in the Supporting Data Values file. The processed RNA-seq data sets in Figure 7, C and D were obtained from an HNSCC cohort (TCGA, Firehose Legacy) and a pan-cancer cohort (ICGC/TCGA, Nature 2020) (23). The processed scRNA-seq data sets in Supplemental Figure 11 were obtained from GEO GSE181919 (24).

## Author contributions

TH, Y Naito, Y Nakanishi, K Masuhiro, MI, TK, SN, JKV, KY, TE, and YH performed the experiments and analyzed the data. M Naito, YM, YY, S Kang, MY, YF, M Nishide, TM, Y Kato, TT, YKK, TA, HT, YT, NT, MT, and HI provided technical support. TH, K Matsuura, and SN independently evaluated the IHC samples. S Koyama and AK conceived the project and supervised the study. All authors contributed to writing the manuscript and discussing the content. TH and Y Naito contributed equally to this work and are co–first authors. TH is listed first because he performed most of the experiments and the integrated analysis. Y Naito is listed next because he performed the core animal experiments and the integrated analysis with TH.

## Supplementary Material

Supplemental data

Supporting data values

## Version 1 02/08/2024

Electronic publication

## References

[1] Bruni D (2020). The immune contexture and immunoscore in cancer prognosis and therapeutic efficacy. Nat Rev Cancer.

[2] Aspeslagh S (2020). Understanding genetic determinants of resistance to immune checkpoint blockers. Semin Cancer Biol.

[3] Joyce JA, Fearon DT (2015). T cell exclusion, immune privilege, and the tumor microenvironment. Science.

[4] Vesely MD (2022). Resistance mechanisms to anti-PD cancer immunotherapy. Annu Rev Immunol.

[5] Spranger S (2015). Melanoma-intrinsic β-catenin signalling prevents anti-tumour immunity. Nature.

[6] Takeuchi Y (2021). Highly immunogenic cancer cells require activation of the WNT pathway for immunological escape. Sci Immunol.

[7] Skoulidis F (2015). Co-occurring genomic alterations define major subsets of KRAS-mutant lung adenocarcinoma with distinct biology, immune profiles, and therapeutic vulnerabilities. Cancer Discov.

[8] Spranger S, Gajewski TF (2018). Impact of oncogenic pathways on evasion of antitumour immune responses. Nat Rev Cancer.

[9] Koyama S (2016). STK11/LKB1 deficiency promotes neutrophil recruitment and proinflammatory cytokine production to suppress T-cell activity in the lung tumor microenvironment. Cancer Res.

[10] Chongsathidkiet P (2018). Sequestration of T cells in bone marrow in the setting of glioblastoma and other intracranial tumors. Nat Med.

[11] Lee JC (2020). Regulatory T cell control of systemic immunity and immunotherapy response in liver metastasis. Sci Immunol.

[12] Yu J (2021). Liver metastasis restrains immunotherapy efficacy via macrophage-mediated T cell elimination. Nat Med.

[13] Kumagai S (2022). Lactic acid promotes PD-1 expression in regulatory T cells in highly glycolytic tumor microenvironments. Cancer Cell.

[14] Nishide M, Kumanogoh A (2018). The role of semaphorins in immune responses and autoimmune rheumatic diseases. Nat Rev Rheumatol.

[15] Nakanishi Y (2021). Neural guidance factors as hubs of immunometabolic cross-talk. Int Immunol.

[16] Naito Y (2023). Tumor-derived semaphorin 4A improves PD-1-blocking antibody efficacy by enhancing CD8^+^ T cell cytotoxicity and proliferation. Sci Adv.

[17] Kinehara Y (2018). Semaphorin 7A promotes EGFR-TKI resistance in EGFR mutant lung adenocarcinoma cells. JCI Insight.

[18] Toyofuku T (2004). Dual roles of Sema6D in cardiac morphogenesis through region-specific association of its receptor, Plexin-A1, with off-track and vascular endothelial growth factor receptor type 2. Genes Dev.

[19] Toyofuku T (2004). Guidance of myocardial patterning in cardiac development by Sema6D reverse signalling. Nat Cell Biol.

[20] Kang S (2018). Semaphorin 6D reverse signaling controls macrophage lipid metabolism and anti-inflammatory polarization. Nat Immunol.

[21] Akbay EA (2017). Interleukin-17A promotes lung tumor progression through neutrophil attraction to tumor sites and mediating resistance to PD-1 blockade. J Thorac Oncol.

[22] Veglia F (2021). Myeloid-derived suppressor cells in the era of increasing myeloid cell diversity. Nat Rev Immunol.

[23] ICGC/TCGA Pan-Cancer Analysis of Whole Genomes Consortium (2020). Pan-cancer analysis of whole genomes. Nature.

[24] Choi JH (2023). Single-cell transcriptome profiling of the stepwise progression of head and neck cancer. Nat Commun.

[25] Gunyuz ZE (2022). SEMA6D differentially regulates proliferation, migration, and invasion of breast cell lines. ACS Omega.

[26] Baxter DE (2021). MiR-195 and its target SEMA6D regulate chemoresponse in breast cancer. Cancers (Basel).

[27] Celus W (2022). Plexin-A4 mediates cytotoxic T-cell trafficking and exclusion in cancer. Cancer Immunol Res.

[28] Takamatsu H (2010). Semaphorins guide the entry of dendritic cells into the lymphatics by activating myosin II. Nat Immunol.

[29] Suto F (2005). Plexin-a4 mediates axon-repulsive activities of both secreted and transmembrane semaphorins and plays roles in nerve fiber guidance. J Neurosci.

[30] Kuwabara S (2021). Microfluidics sorting enables the isolation of an intact cellular pair complex of CD8^+^ T cells and antigen-presenting cells in a cognate antigen recognition-dependent manner. PLoS One.

[31] Aoshi T (2005). Expression mapping using a retroviral vector for CD8^+^ T cell epitopes: definition of a Mycobacterium tuberculosis peptide presented by H2-Dd. J Immunol Methods.

[32] Naito M (2022). Semaphorin 6D-expressing mesenchymal cells regulate IL-10 production by ILC2s in the lung. Life Sci Alliance.

[33] Nakatani T (2021). The lysosomal Ragulator complex plays an essential role in leukocyte trafficking by activating myosin II. Nat Commun.

[34] Hao Y (2021). Integrated analysis of multimodal single-cell data. Cell.

[35] Korsunsky I (2019). Fast, sensitive and accurate integration of single-cell data with Harmony. Nat Methods.

