# Supplementary material for: Sema6D forward signaling impairs T cell activation and proliferation in head and neck cancer
Source: JCI Insight. 2024 Feb 8;9(3):e166349. doi: 10.1172/jci.insight.166349 (PMC10967380; doi:10.1172/jci.insight.166349)
Supplement: Supplemental data [file jciinsight-9-166349-s019.pdf]

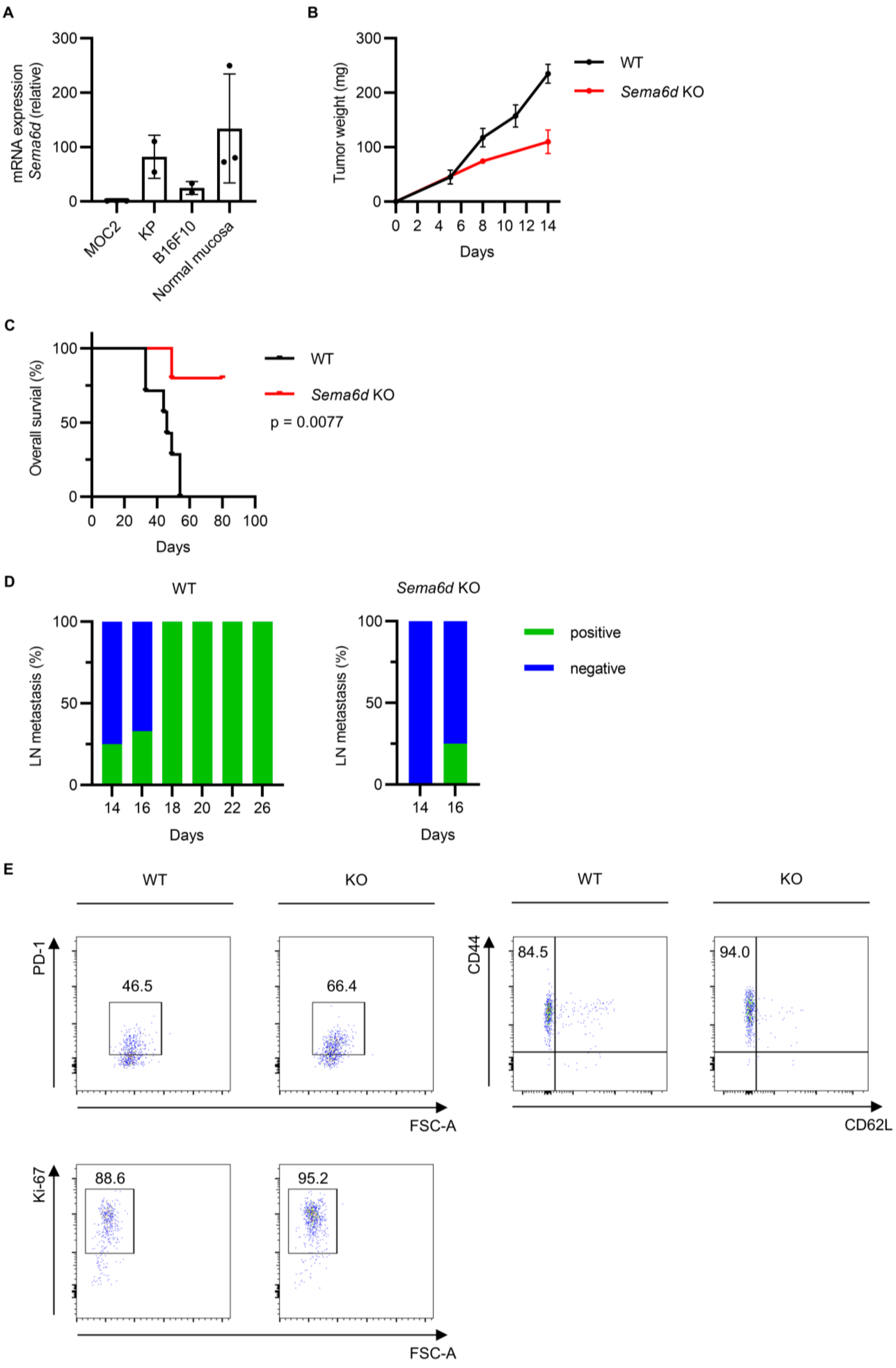
A
mRNA expression
Sema6d (relative)
300
200
100
0
MOC2
KP
B16F10
Normal mucosa
B
Tumor weight (mg)
Days
WT
Sema6d KO
C
Overall survial (%)
Days
WT
Sema6d KO
p = 0.0077
D
WT
Sema6d KO
LN metastasis (%)
Days
positive
negative
E
WT
KO
PD-1
46.5
66.4
FSC-A
CD44
84.5
94.0
CD62L
Ki-67
88.6
95.2
FSC-A

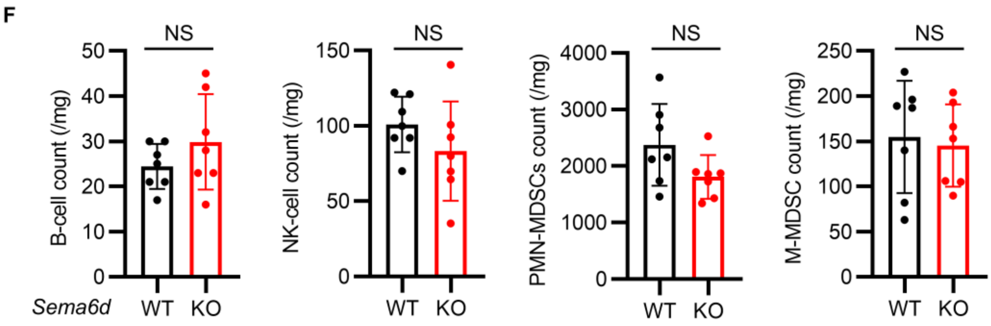

Supplemental Figure 1. Phenotypic differences between WT and *Sema6d* KO mice in a murine oral cancer model.

(A) Expression of *Sema6d* in various cancer cell lines and normal oral mucosa in mice was evaluated by quantitative PCR (n = 2–3 for each group). (B) Tumor growth curves of WT (n = 4) vs *Sema6d* KO mice (n = 4) administered MOC2 cells. (C) Kaplan-Meier survival curves of WT (n = 7) vs *Sema6d* KO mice (n = 5) administered MOC2 cells. P = 0.0077; statistical significance determined by log-rank test. Data are representative of two independent experiments. (D) Percentage of tumor-draining lymph node metastases and days after MOC2 cell administration in WT and *Sma6d* KO mice. (WT: n = 34, *Sema6d* KO: n = 10). (E) Representative flow cytometry data of the frequencies of PD-1, CD44, CD62L, and Ki-67 positivity in tumor-infiltrating $CD8^+$ T cells in WT vs *Sema6d* KO mice at day 14 after MOC2 injection. (F) B cells, NK cells, M-MDSCs, and PMN-MDSCs in the TME were compared in WT (n = 7) vs *Sema6d* KO mice (n = 7) at day 14 after MOC2 cell injection. Data are representative of two independent experiments. NS: not significant; statistical significance determined by Student's *t* test. Results are presented as the mean ± SEM.

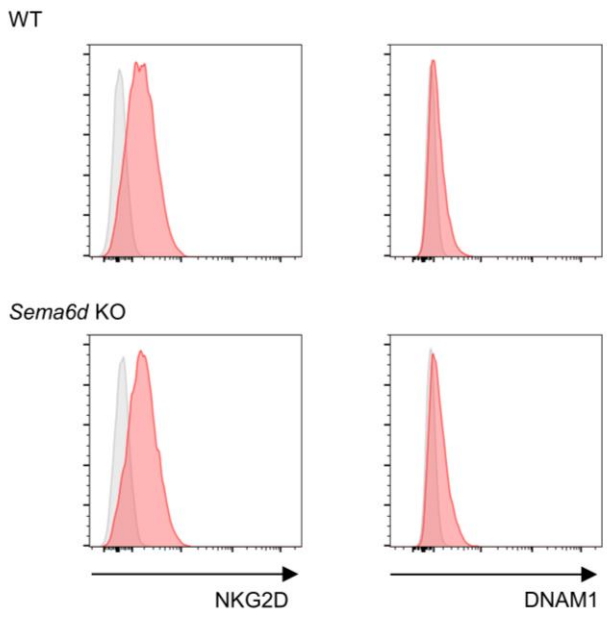

Supplemental Figure 2. Sema6D is dispensable for NK-cell activation.

The expression of the activating receptors NKG2D and DNAM1 was compared in NK cells from splenocytes in WT and *Sema6d* KO mice by flow cytometry. Gray histograms indicate isotype control. Data are representative of three independent experiments.

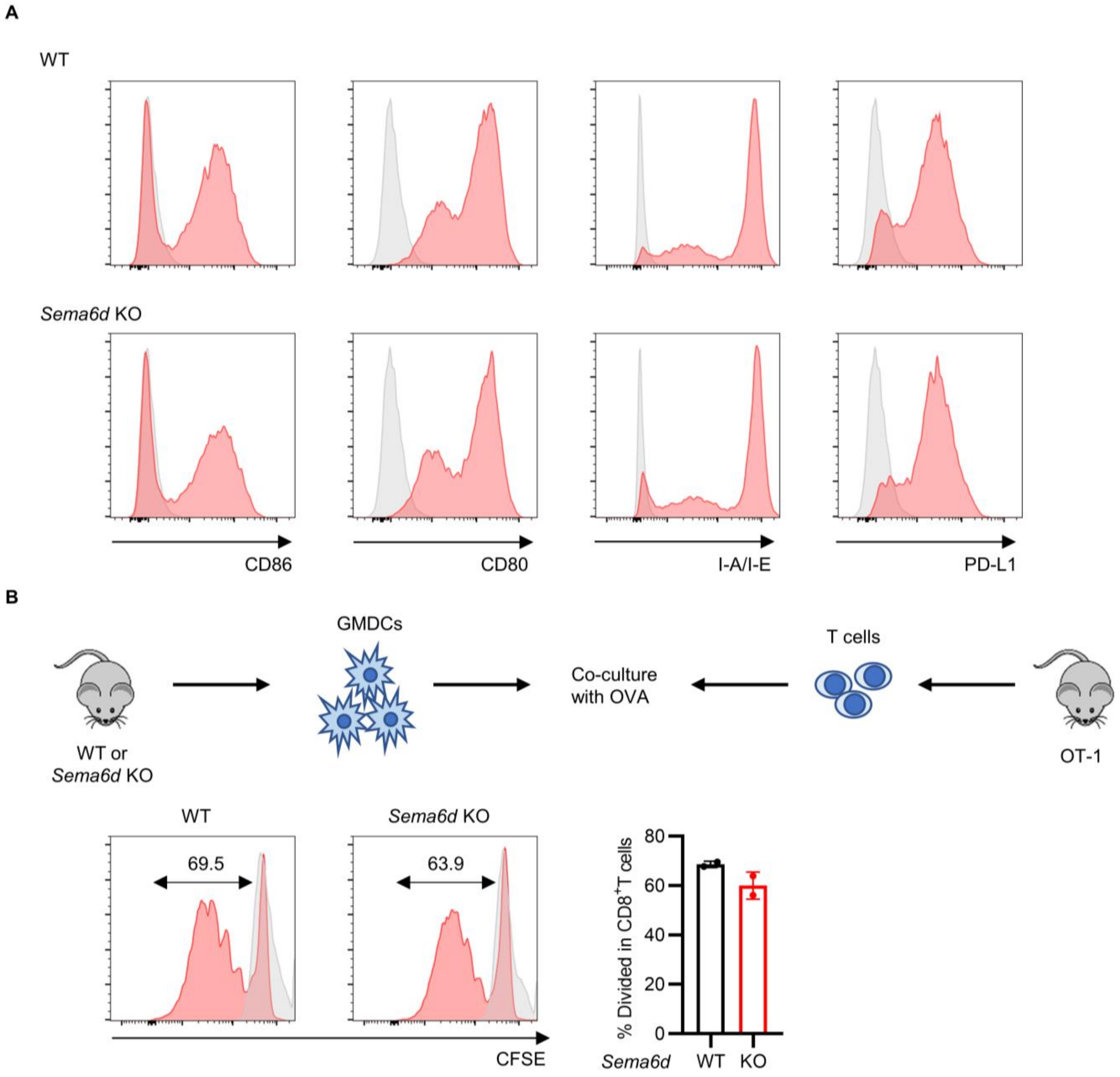

Supplemental Figure 3. Sema6D is dispensable for maturation, activation, and antigen-presenting ability of dendritic cells (DCs).

(A) The expression of maturation markers (CD86, CD80) and activation markers (I-A/I-E, PD-L1) in bone marrow-derived DCs developed by GM-CSF (GMDCs) from WT and *Sema6d* KO mice was evaluated by flow cytometry. Gray histograms indicate isotype control. Data are representative of two independent experiments. (B) GMDCs from WT and *Sema6d* KO mice and T cells from OT-1 mice were co-cultured with OVA peptide. $CD8^+$ T-cell proliferation was evaluated after 72 hours (WT: n = 2, *Sema6d* KO: n = 2). Representative histograms of $CD8^+$ T-cell proliferation are shown. Data are representative of three independent experiments. Results are presented as the mean ± SEM.

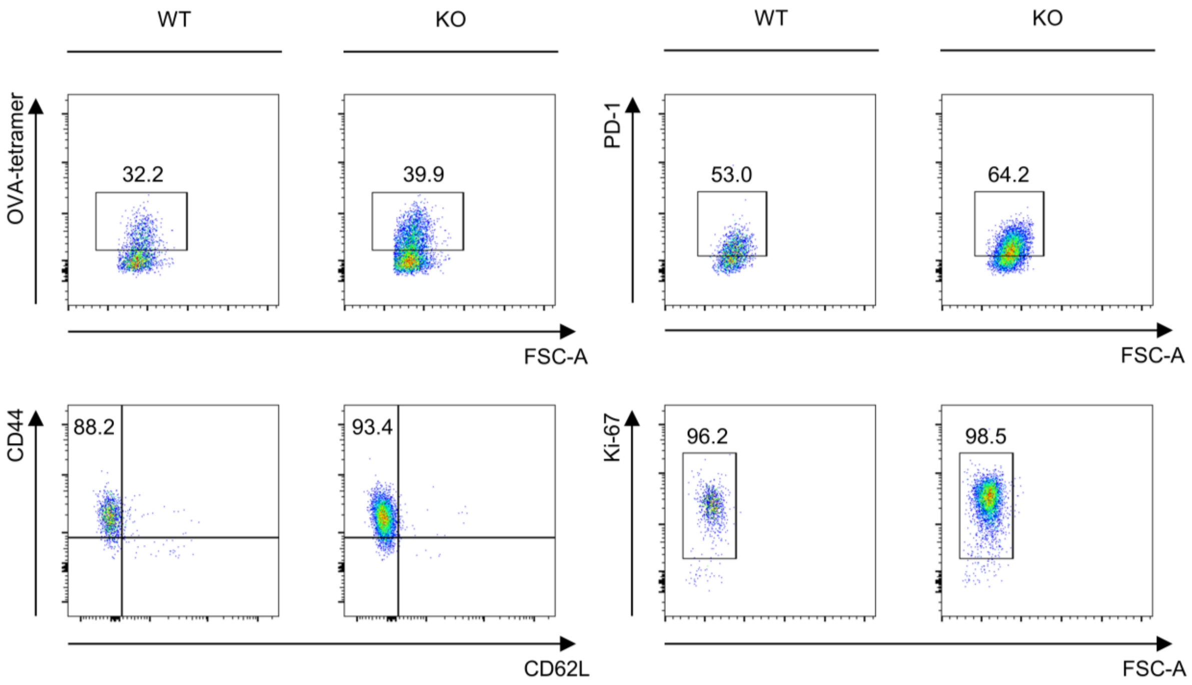

Supplemental Figure 4. Representative flow cytometry data of the frequencies of OVA-tetramer, PD-1, CD44, CD62L, and Ki-67 positivity in tumor-infiltrating CD8^{+} T cells in WT vs *Sema6d* KO mice at day 14 after MOC2^{OVA} injection.

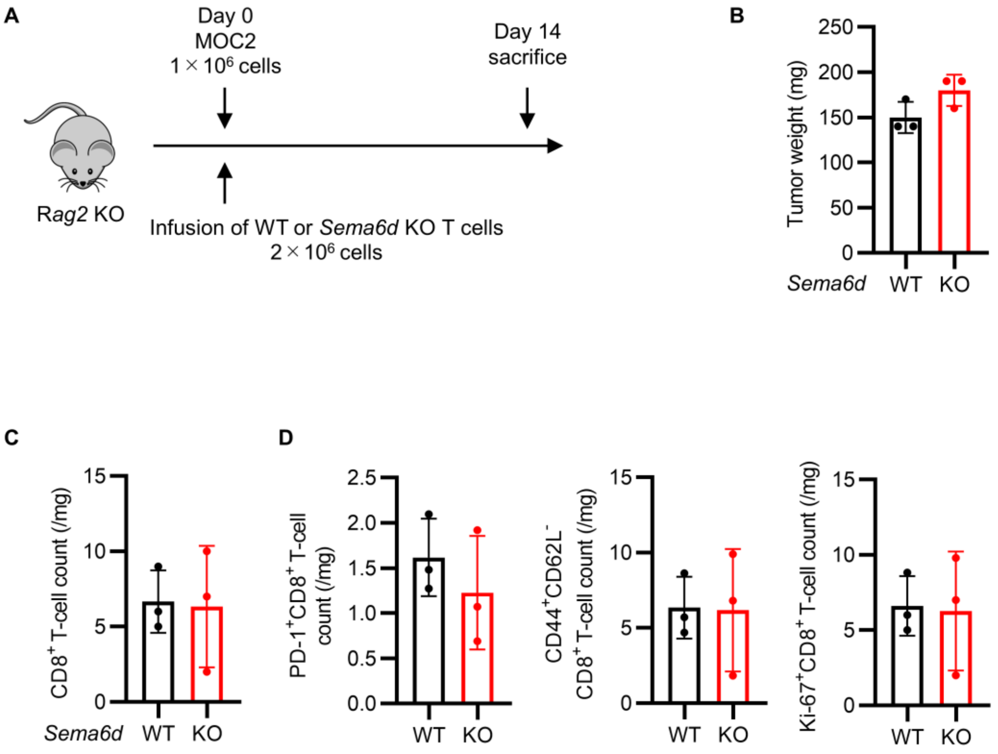

Supplemental Figure 5. Sema6D in T cells is not involved in these cells' migration into tumors or their anti-tumor function.

(A) Schematic for immunological analysis of *Rag2* KO mice administered either WT or *Sema6d* KO T cells and MOC2 cells. (B) Tumor weight at day 14 after administration of WT (n = 3) or *Sema6d* KO T cells (n = 3). Results are expressed as the mean ± SEM. (C) $CD8^+$ T-cell count and (D) activation and differentiation markers of tumor-infiltrating $CD8^+$ T cells at day 14 after T-cell infusion (WT: n = 3 and *Sema6d* KO: n = 3) were analyzed by flow cytometry. (B, C, and D) Data are representative of two independent experiments. Results are expressed as the mean ± SEM.

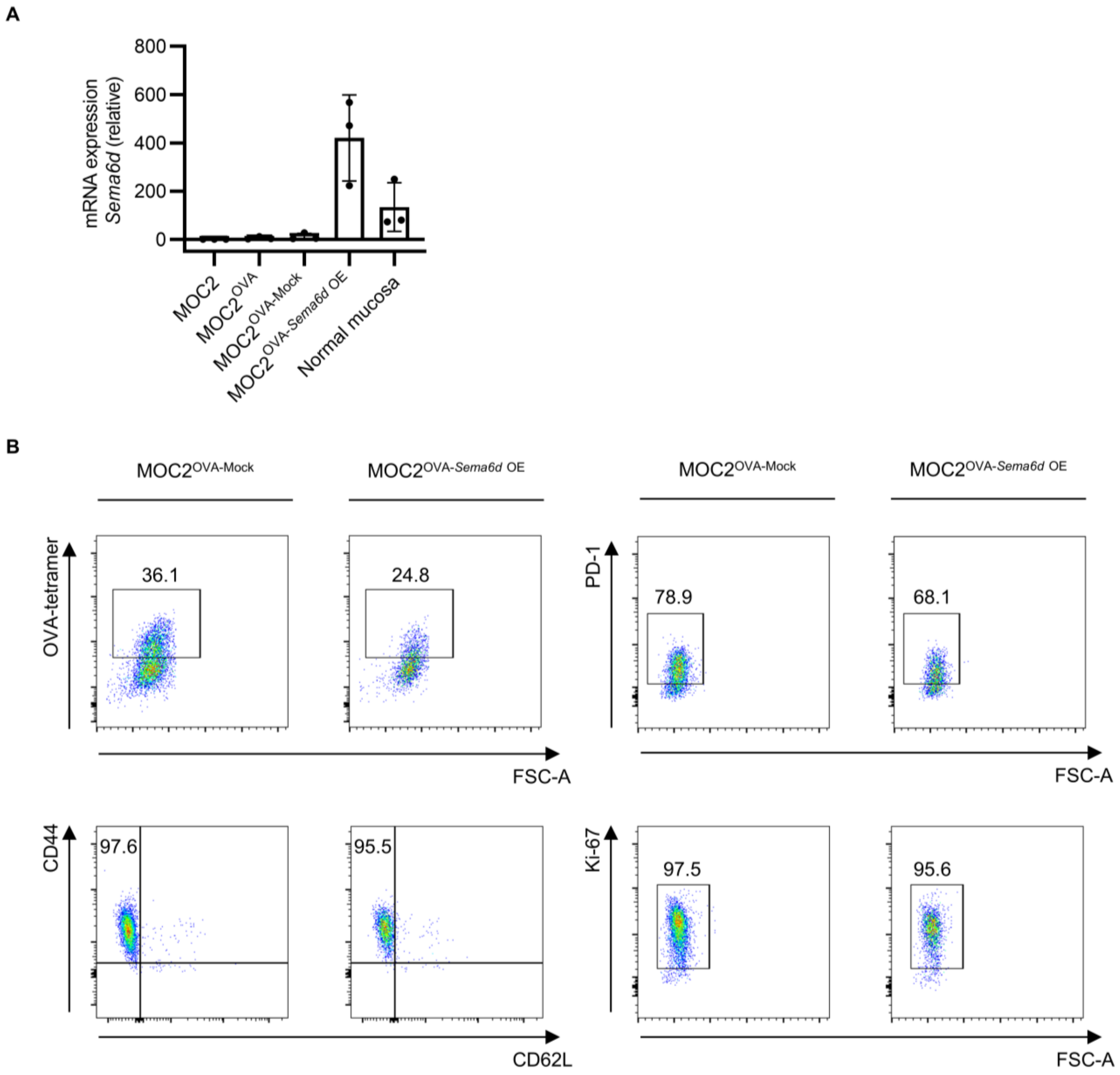

Supplemental Figure 6. Expression of *Sema6d* in cell lines and normal oral mucosa, and representative flow cytometry data of infiltrating CD8^{+} T cells injected MOC2$^{\text{OVA-Mock}}$ or MOC2$^{\text{OVA-}\textit{Sema6d}\text{ OE}}$ cells.

(A) Expression of *Sema6d* in MOC2, MOC2^{OVA}, MOC2$^{\text{OVA-Mock}}$, MOC2$^{\text{OVA-}\textit{Sema6d}\text{ OE}}$ cell lines and normal oral mucosa in mice (n = 3 each group) was evaluated by quantitative PCR. Data are representative of two independent experiments. Results are expressed as the mean ± SEM. (B) Representative flow cytometry data of the frequencies of OVA-tetramer, PD-1, CD44, CD62L, and Ki-67 positivity in tumor-infiltrating CD8^{+} T cells at day 14 after MOC2$^{\text{OVA-Mock}}$ or MOC2$^{\text{OVA-}\textit{Sema6d}\text{ OE}}$ injection.

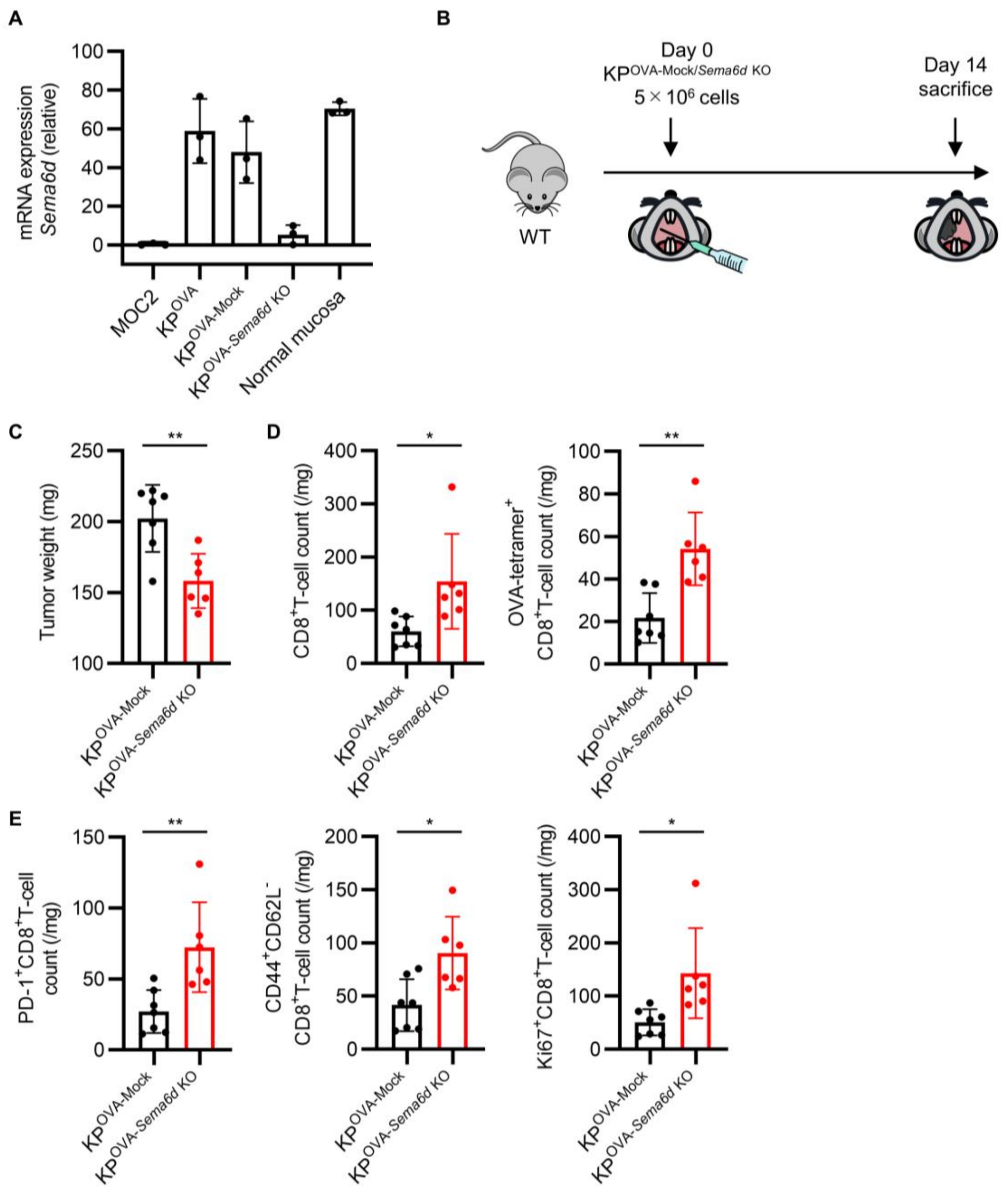

Supplemental Figure 7. Removal of Sema6D of tumor cells improves CD8^{+} T-cell activation and proliferation in the TME.

(A) Expression of *Sema6d* in MOC2, KP^{OVA}, KP$^{OVA\text{-}Mock}$, KP$^{OVA\text{-}\mathit{Sema6d}\ KO}$ cell lines and normal oral mucosa in mice (n = 3 each group) was evaluated by quantitative PCR. Data are representative of two independent experiments. Results are expressed as the mean ± SEM. (B) Schematic of KP$^{OVA\text{-}Mock}$ or KP$^{OVA\text{-}\mathit{Sema6d}\ KO}$ cell injection and immunological analysis in WT mice. (C) Tumor weight at day 14 after administration of KP$^{OVA\text{-}Mock}$ (n = 7) or KP$^{OVA\text{-}\mathit{Sema6d}\ KO}$ cells (n = 6). (D) CD8^{+} T-cell counts and OVA-tetramer^{+}CD8^{+} T-cell counts in TME, and (E) activation and differentiation markers of tumor-infiltrating CD8^{+} T cells at day 14 after KP$^{OVA\text{-}Mock}$ (n = 7) or KP$^{OVA\text{-}\mathit{Sema6d}\ KO}$ cells (n = 6) injection were analyzed by flow cytometry. (C, D, and E) Data are representative of two independent experiments. *$P < 0.05$, **$P < 0.01$; statistical significance determined by Student's *t* test. Results are expressed as the mean ± SEM.

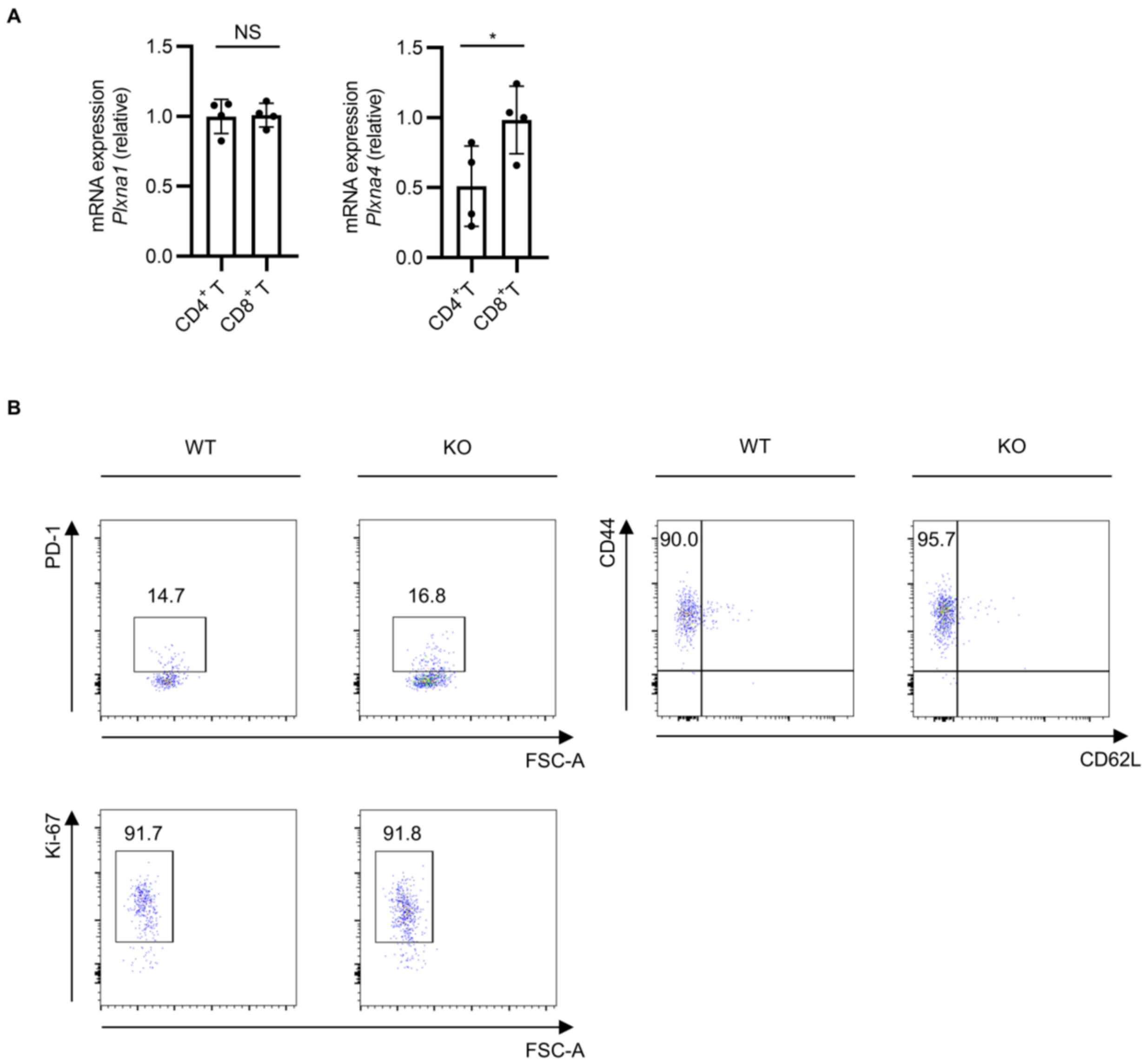

Supplemental Figure 8. Expression of *Plxna1* and *Plxna4* in CD4$^+$ T cells and CD8$^+$ T cells from tumor-draining lymph nodes, and representative flow cytometry data of infiltrating CD8$^+$ T cells in WT vs *Plxna4* KO mice.

(A) Expression of *Plxna1* and *Plxna4* in CD4$^+$ T cells and CD8$^+$ T cells isolated from tumor-draining lymph nodes was evaluated by quantitative PCR (n = 4 each group). Data are representative of two independent experiments. NS: not significant, *P < 0.05; statistical significance determined by Student's *t* test. Results are expressed as the mean ± SEM. (B) Representative flow cytometry data of the frequencies of PD-1, CD44, CD62L, and Ki-67 positivity in tumor-infiltrating CD8$^+$ T cells in WT vs *Plxna4* KO mice at day 14 after MOC2 injection.

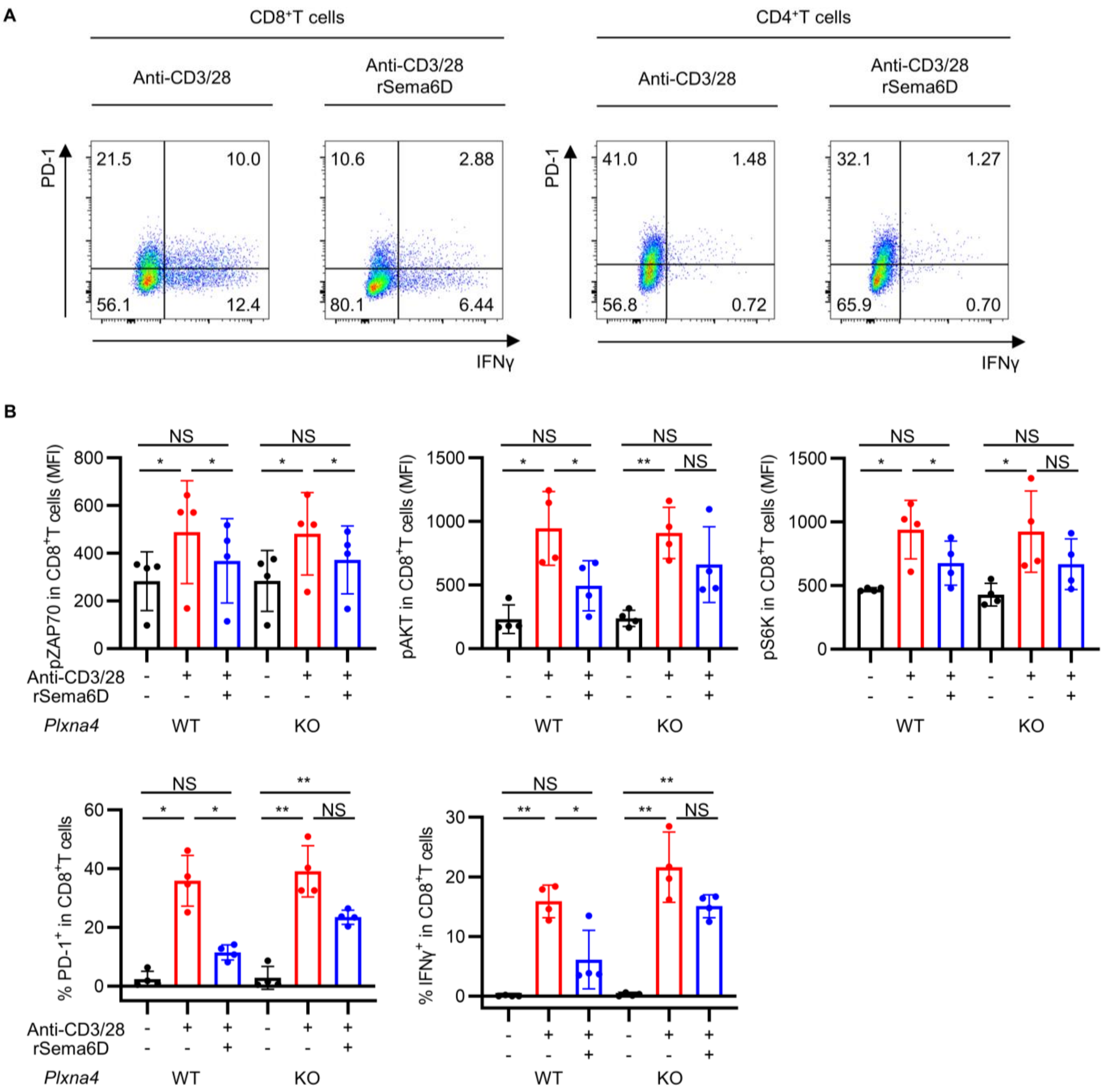

Supplemental Figure 9. Sema6D suppresses activation of CD8^{+} T cells in WT, but the suppression is partially restored in CD8^{+} T cells in *Plxna4* KO mice.

(A) Representative flow cytometry data of PD-1 and IFNγ of CD8^{+} T cells and CD4^{+} T cells stimulated with or without rSema6D in CD8^{+} and CD4^{+} T cells isolated from tumor-draining lymph nodes of WT mice. (B) Phosphorylation of ZAP70 (pZAP70), AKT (pAKT), S6-kinase (pS6K), and positivity for PD-1 and IFNγ in CD8^{+} T cells isolated from tumor-draining lymph nodes of WT or *Plxna4* KO mice and stimulated as follows: no stimulation, anti-CD3/28 antibody (48 hours), and anti-CD3/28 antibody plus rSema6D (48 hours) (n = 4 each group). Data are representative of two independent experiments. NS: not significant, *P < 0.05, **P < 0.01; statistical significance determined by one-way ANOVA. Results are expressed as the mean ± SEM.

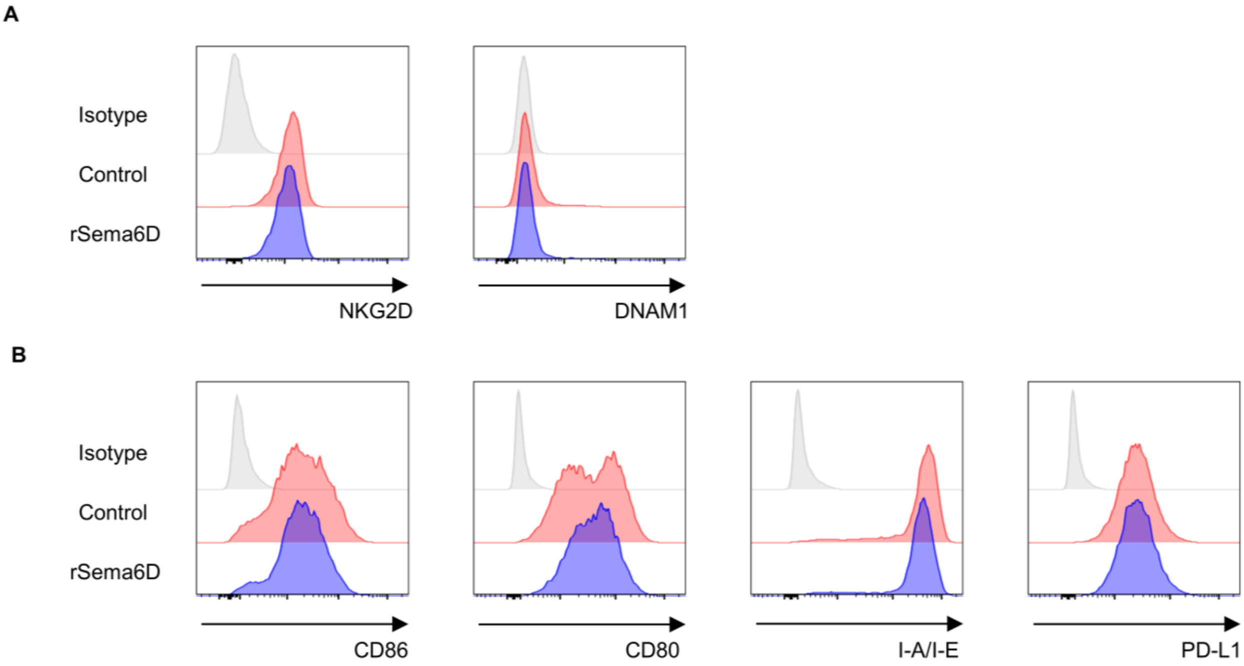

Supplemental Figure 10. rSema6D stimulation does not change the activation or maturation of NK cells or DCs.

(A, B) NK cells from splenocytes (A) and GMDCs (B) were cultured with or without rSema6D for 24 hours. Activating receptors of NK cells (A) and maturation and activation markers in GMDCs (B) were analyzed by flow cytometry. Gray histograms: isotype control, red histograms: control, and blue histograms: rSema6D.

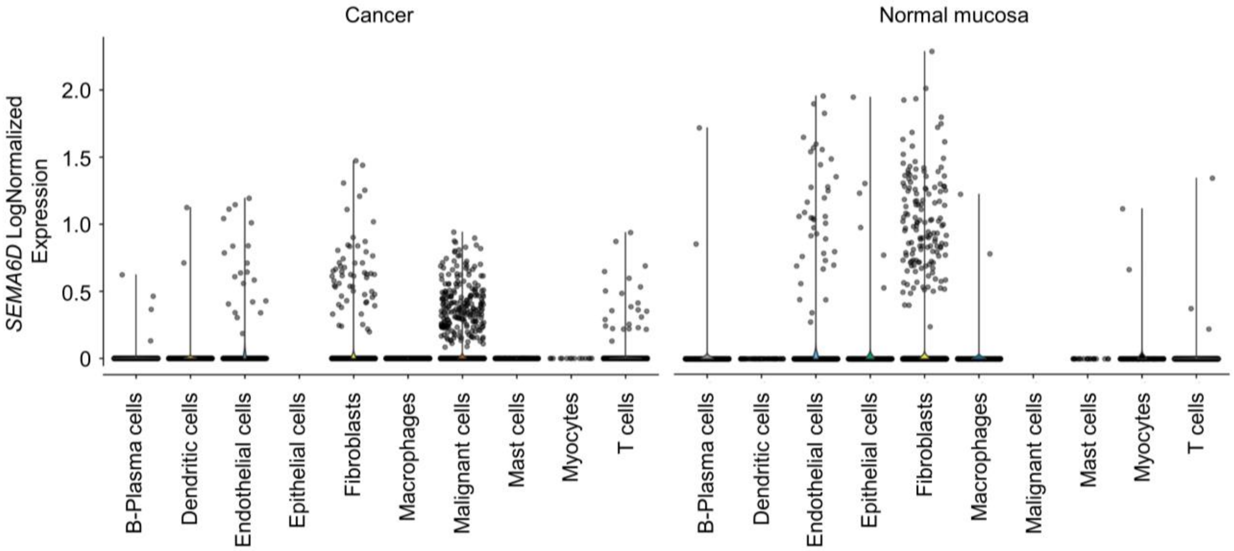

Supplemental Figure 11. Expression of *SEMA6D* in each cell of human oral cancer and normal oral mucosa derived from single cell RNA sequence (n = 23; 54,239 total single cells).

This figure was analyzed from public single cell RNA sequence data.

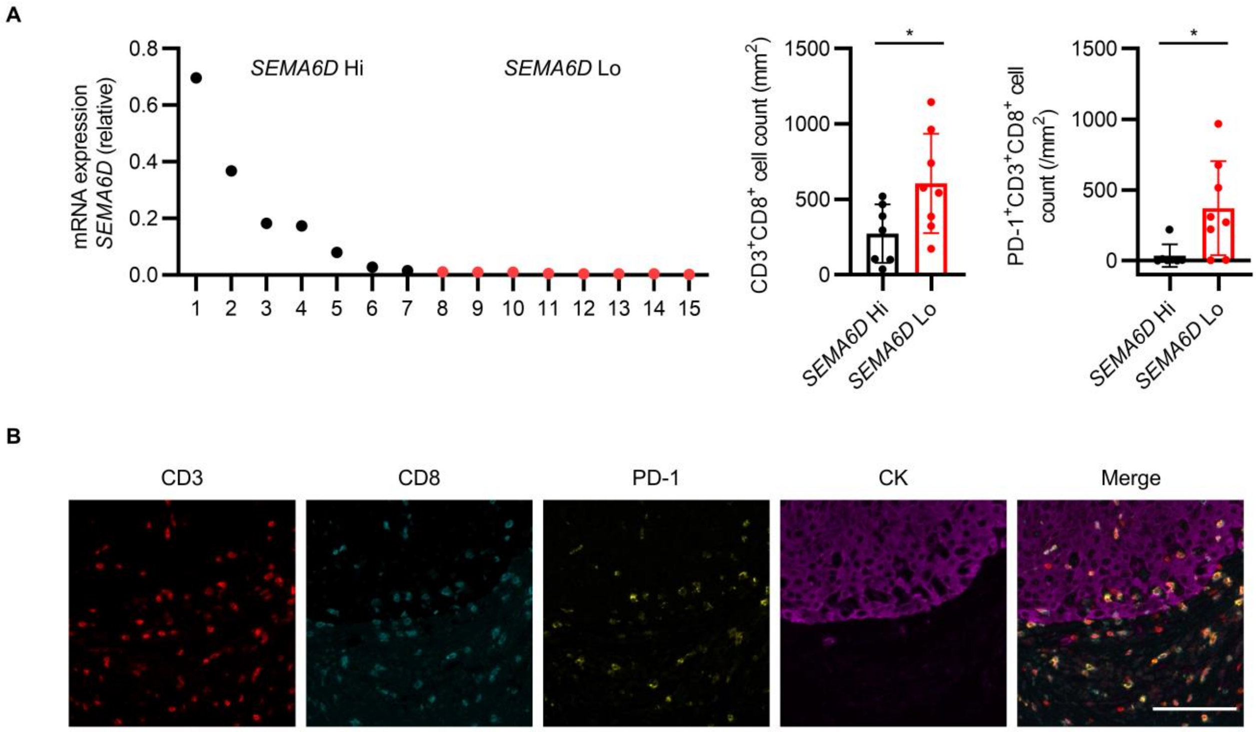

Supplemental Figure 12. CD3$^+$CD8$^+$ cell counts and PD-1$^+$CD3$^+$CD8$^+$ cell counts in tumors were increased in *SEMA6D* low HNSCC.

(A) Expression of *SEMA6D* in HNSCC was evaluated by quantitative PCR (n = 15). CD3$^+$CD8$^+$ cell counts and PD-1$^+$CD3$^+$CD8$^+$ cell counts in tumors were compared between *SEMA6D* high tumor (n = 7) and *SEMA6D* low tumor (n = 8). *P < 0.05; statistical significance determined by Student's *t* test. Results are expressed as the mean ± SEM. (B) Representative images of fluorescence immunohistochemical staining for CD3, CD8, PD-1, Cytokeratin (CK), Merge in tumors. Magnification = 200x. Scale bar: 100 µm.

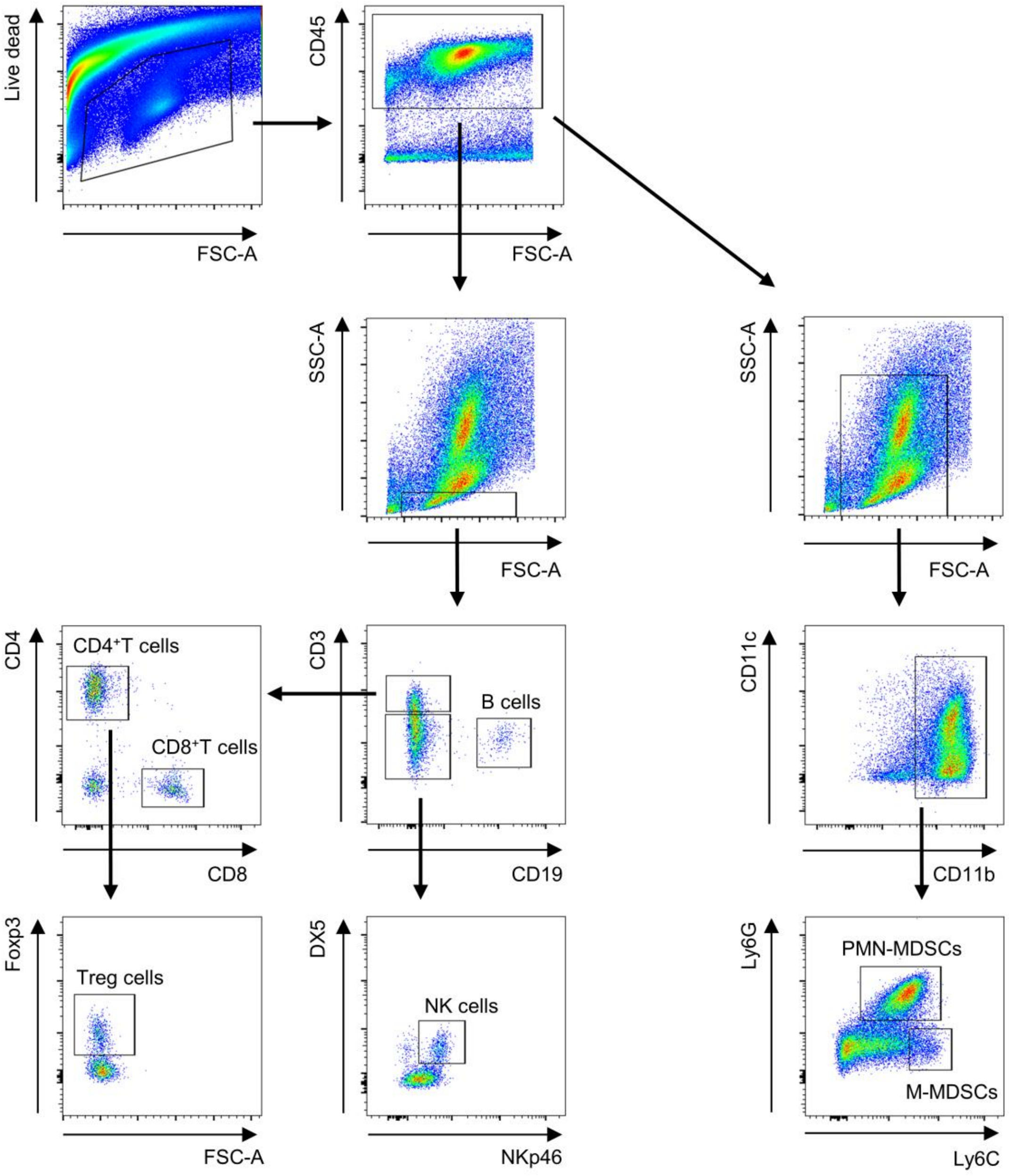

Supplemental Method

Gating strategy for flow cytometry analysis. Defined CD4$^+$ T cells, CD8$^+$ T cells, B cells, Treg cells, NK cells, PMN-MDSCs, and M-MDSCs as shown.

Immunohistochemistry antibody list (mouse)

Molecule	Clone	Manufacturer
CD8a	D4W2Z	Cell Signaling Technology

Immunohistochemistry antibody list (human)

Molecule	Clone	Manufacturer
CD3	SP162	Abcam
CD8a	C8/144B	Abcam
Cytokeratin	AE1/AE3	Abcam
PD-1	EPR4877	Abcam

Flow cytometry antibody list

Molecule	Clone	Fluorescence	Manufacturer
CD3e	145-2C11	FITC	BD Biosciences
	145-2C11	Brilliant Violet 421	BD Biosciences
	145-2C11	PE/Cyanine7	BioLegend
	17A2	APC/Cyanine7	BioLegend
CD4	RM4-5	APC	BioLegend
	RM4-5	Pacific Blue	BD Biosciences
	GK1.5	PE/Cyanine7	BioLegend
	RM4-5	APC/Cyanine7	BioLegend
CD8a	53-6.7	PE	eBioscience
	KT15	FITC	MBL
	53-6.7	PerCP/Cyanine5.5	BioLegend
	53-6.7	Brilliant Violet 421	BioLegend
	53-6.7	APC/Cyanine7	BioLegend
CD11b	M1/70	PE/Cyanine7<br>APC/Cyanine7	BioLegend
CD11c	N418	Brilliant Violet 421	BioLegend
CD19	6D5	Brilliant Violet 421	BioLegend
CD40 / Isotype	3/23 / R35-95	FITC	BD Biosciences
CD44 / Isotype	IM7 / RTK4530	PE<br>PerCP/Cyanine5.5	BioLegend

CD45	30-F11	Alexa Fluor® 488<br>PE/Cyanine7<br>APC/Cyanine7	BioLegend
CD49b (pan-NK cells)	DX5	Alexa Fluor® 488	BioLegend
CD62L / Isotype	MEL-14 / RTK2758	APC	BioLegend
CD80 / Isotype	16-10A1 / HTK888	PE	BioLegend
CD86 / Isotype	GL1 / R35-95	FITC	BD Biosciences
CD226 (DNAM-1) / Isotype	10E5 / RTK4530	PE	BioLegend
CD274 (PD-L1) / Isotype	10F.9G2 / RTK4530	PE	BioLegend
CD279 (PD-1) / Isotype	29F.1A12 / RTK2758	FITC<br>PerCP/Cyanine5.5	BioLegend
CD314 (NKG2D) / Isotype	CX5 / RTK2071	APC	BioLegend
CD335 (Nkp46)	29A1.4	PerCP/Cyanine5.5	BioLegend
Foxp3 / Isotype	FJK-16s / eBR2a	APC	eBioscience
IFN gamma / Isotype	XMG1.2 / eBRG1	eFluor 450	eBioscience
Ki-67 / Isotype	16A8 / RKT2758	APC<br>Brilliant Violet 421	BioLegend
Ly-6C	HK1.4	FITC	BioLegend
Ly-6G	1A8	PerCP/Cyanine5.5	BioLegend
MHC Class II(I-A/I-E) / Isotype	M5/114.15.2 / RTK4530	PerCP/Cyanine5.5	BioLegend
AKT pS473 / Isotype	REA359 / REA293	APC	Miltenyi Biotec
p70 S6 Kinase / Isotype	REA843 / REA293	FITC	Miltenyi Biotec
pZAP70 Syk (Tyr319, Tyr352) / Isotype	n3kobu5 / eBMG2b	PE	eBioscience
Siglec-F	E50-2440	Alexa Fluor® 647	BD Biosciences
T-Select H-2K^{b}OVA Tetramer-SIINFEKL / Isotype	-	PE	MBL
